# Proteomic Screening for Amyloid Proteins

**DOI:** 10.1371/journal.pone.0116003

**Published:** 2014-12-30

**Authors:** Anton A. Nizhnikov, Alexander I. Alexandrov, Tatyana A. Ryzhova, Olga V. Mitkevich, Alexander A. Dergalev, Michael D. Ter-Avanesyan, Alexey P. Galkin

**Affiliations:** 1 Dept. of Genetics and Biotechnology, St. Petersburg State University, St. Petersburg, Russia; 2 St. Petersburg Branch, Vavilov Institute of General Genetics of the Russian Academy of Sciences, St. Petersburg, Russia; 3 A.N. Bach Institute of Biochemistry of the Russian Academy of Sciences, Moscow, Russia; University of Maryland School of Medicine, United States of America

## Abstract

Despite extensive study, progress in elucidation of biological functions of amyloids and their role in pathology is largely restrained due to the lack of universal and reliable biochemical methods for their discovery. All biochemical methods developed so far allowed only identification of glutamine/asparagine-rich amyloid-forming proteins or proteins comprising amyloids that form large deposits. In this article we present a proteomic approach which may enable identification of a broad range of amyloid-forming proteins independently of specific features of their sequences or levels of expression. This approach is based on the isolation of protein fractions enriched with amyloid aggregates via sedimentation by ultracentrifugation in the presence of strong ionic detergents, such as sarkosyl or SDS. Sedimented proteins are then separated either by 2D difference gel electrophoresis or by SDS-PAGE, if they are insoluble in the buffer used for 2D difference gel electrophoresis, after which they are identified by mass-spectrometry. We validated this approach by detection of known yeast prions and mammalian proteins with established capacity for amyloid formation and also revealed yeast proteins forming detergent-insoluble aggregates in the presence of human huntingtin with expanded polyglutamine domain. Notably, with one exception, all these proteins contained glutamine/asparagine-rich stretches suggesting that their aggregates arose due to polymerization cross-seeding by human huntingtin. Importantly, though the approach was developed in a yeast model, it can easily be applied to any organism thus representing an efficient and universal tool for screening for amyloid proteins.

## Introduction

An increasing amount of findings demonstrates that amyloids can be a functional form of a protein in a broad range of organisms from bacteria to mammals. However, interest in such protein aggregates is still mainly caused by their relation to human and animal pathologies. To date, more than 30 amyloids related to human disorders have been identified [1]. The most common among these disorders are Alzheimer, Parkinson, Huntington diseases, type 2 diabetes mellitus, and some forms of spinocerebellar ataxia and cataract [2], [3]. A distinct group of these disorders are prion diseases caused by an infectious amyloid form of the prion protein, called PrP^Sc^ [4]. Self-perpetuating prion amyloids have also been described in lower eukaryotes, yeast *Saccharomyces cerevisiae* [5]–[14] and filamentous fungus *Podospora anserina* [15], in which they determine non-chromosomally inherited phenotypes. Notably, in spite of significant progress in discovering amyloids, most of them were found by analyzing proteins with sequences similar to known amyloid-forming proteins [6], [9] or via time-consuming and sometimes highly sophisticated genetic screenings for factors that determine non-Mendelian traits [7], [8], [11], [12]. This suggests that development of a reliable and universal biochemical approach for fast identification of novel amyloid-forming proteins is both important and timely.

To date, biochemical approaches used to identify novel amyloid proteins have been based on their common ability to form insoluble aggregates. For example, the prion form of PrP was detected as a major protease-resistant protein component of infectious, high molecular weight aggregates isolated by ultracentrifugation [16]. Amyloid beta peptide (Aβ) was extracted from a protein pellet fraction obtained from brain homogenate of a patient, who had died of Alzheimer’s disease [17]. Unfortunately, such approaches can only be used for identification of amyloids that form large deposits.

A common property of amyloid aggregates is resistance to various detergents [18]–[20]. Amyloid nature of such detergent-insoluble aggregates is supported by the fact that they often contain generic amyloid epitopes for DNA aptamer binding [21]. High resistance of amyloids to treatment with detergents allows amyloid isolation from yeast cell lysates by sedimentation in the presence of SDS [22]. A validation of this method carried out for the yeast prion [*PSI* ^+^] (a prion form of Sup35), demonstrated its suitability for identification of proteins forming relatively abundant amyloids. An alternative approach based on separation of SDS-insoluble amyloids by their inability to enter polyacrylamide gel and subsequent mass-spectrometry allowed the identification of the Rnq1 and Ure2 prion proteins, which are less abundant than Sup35 [23]. Nevertheless, both these methods enable only identification of proteins comprising amyloids that are resistant to treatment with SDS, while some amyloids, which are not enriched in Q or N residues, such as amyloids of Aβ(1-40aa), are soluble in the presence of SDS [24].

Here, we present a novel proteomic approach for screening for amyloid proteins, called PSIA (Proteomic Screening for Identification of Amyloid proteins). This approach can use either SDS or sarkosyl for amyloid isolation. We show that the use of sarkosyl (Sodium N-lauroylsarcosinate) instead of SDS for the purification of amyloid aggregates allows isolation of amyloids which cannot withstand SDS treatment, thus making the approach more universal than those which were developed earlier. This approach was validated in a yeast model by detection of prion proteins and mammalian amyloid-forming proteins. We also identified several yeast proteins which form detergent-insoluble aggregates in response to expression of human huntingtin with an expanded polyglutamine domain.

## Results

### Identification of yeast prion and mammalian amyloid proteins

**Figure 1:**
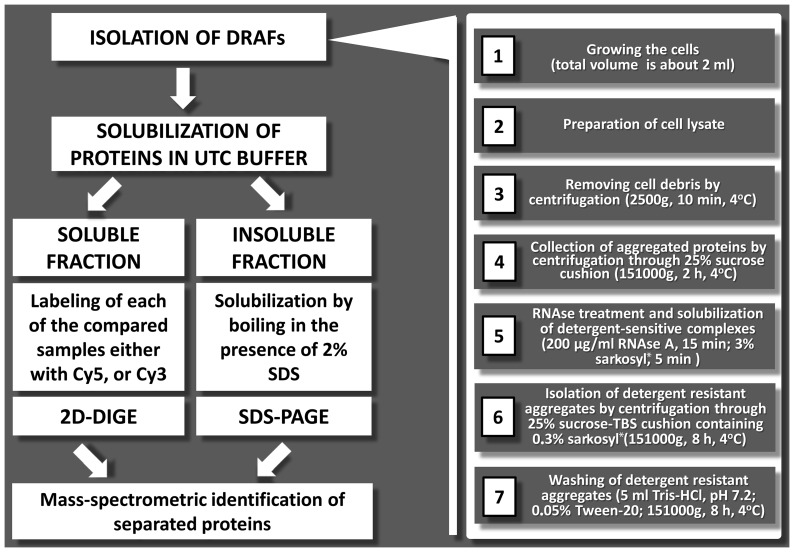
Schematic representation of PSIA. Left panel illustrates the main stages of PSIA. Right panel describes the procedures for DRAF isolation. For details, see Materials and methods. *1% and 0.1% SDS can be used instead of 3% and 0.3% sarkosyl in steps 5 and 6, respectively.

PSIA approach developed in this study consists of three general steps: isolation of detergent-resistant aggregate fractions (DRAFs), separation of proteins from DRAFs and identification of separated proteins. The principal scheme of the approach is shown in Fig. 1. Successive stages of PSIA are described in the corresponding sections of Materials and Methods.

**Figure 2:**
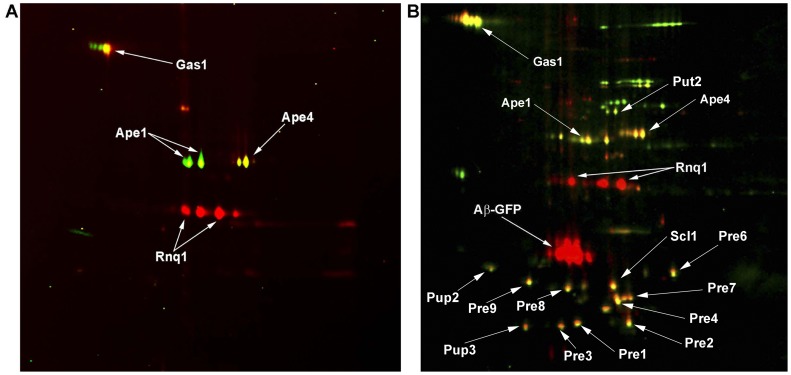
2D-DIGE image of proteins of SDS- (A) or sarkosyl-insoluble (B) aggregates isolated from the BY4742 [*PIN* ^+^] strain expressing Aβ-GFP and from its [*pin* ^−^] derivative expressing non-fused GFP. Spots corresponding to proteins from [*PIN* ^+^] cells expressing Aβ-GFP are red, while proteins from GFP-containing cells (control) are green. Yellow spots correspond to proteins present in both compared samples. Proteins identified by mass-spectrometry are indicated. Identification data are presented in Table 1 (SDS-insoluble aggregates) and 2 and 3(sarkosyl-insoluble aggregates). Mass-spectra of identified proteins are listed in S1–S3 Tables, correspondingly.

First, we tested the efficiency of our approach for the detection of the Rnq1 protein which is the structural determinant of the yeast prion [*PIN* ^+^] [7] and for human Aβ (1–40 aa) peptide tagged with GFP (designated as Aβ-GFP further in the text), which is known to form amyloid-like aggregates in yeast cells both in the presence and absence of [*PIN* ^+^] [25]. DRAFs from the BY4742 [*PIN* ^+^] strain expressing Aβ-GFP and from its [*pin* ^−^] derivative expressing non-fused GFP protein were isolated using 1% SDS. The DRAFs from test and control samples were solubilized in UTC buffer (8 M urea, 2 M thiourea, 4% CHAPS, and 30 mM TrisHCl pH 8.5). Proteins from [*PIN* ^+^]/Aβ-GFP and [*pin* ^−^]/GFP strains were labeled with Cy5 and Cy3 fluorescent dyes, correspondingly, then the samples were mixed and analyzed by two-dimensional difference gel electrophoresis (2D-DIGE) (Fig. 2A) [26]. Proteins present only in the test sample ([*PIN* ^+^]/Aβ-GFP) are pseudocolored in red, while proteins from the control sample ([*pin* ^−^]/GFP) are pseudocolored in green. Yellow spots correspond to proteins present in both the test and control samples. Interestingly, mass-spectrometric analysis showed that the red spots correspond to Rnq1 (Fig. 2A, Table 1 and S1 Table). Multiple spots corresponding to Rnq1 may appear due to its slight proteolysis or differential post-translational modification, such as phosphorylation. Identification of Rnq1 shows that the approach is relatively sensitive, since Rnq1 is not an abundant protein (∼1000 molecules of protein per cell) [27] **.** We also identified Ape1, Ape4 and Gas1 proteins presented in both [*PIN* ^+^]/Aβ-GFP and [*pin* ^−^]/GFP strains (yellow spots) (Fig. 2A, Table 1 and S1 Table). Notably, no red spots corresponded to Aβ-GFP, confirming previous data, which indicate that treatment with SDS solubilizes aggregates of this protein [24].

**Table 1:** Identification of proteins in DRAFs isolated by treatment with SDS from the BY4742 [*PIN* ^+^] strain and its [*pin* ^−^] derivative.

Accession^a^	Protein b	Score^c^	Molecularmass	Calculatedisoelectric point	Peptidecount^d^	Coverage^e^
6322746	Ape1	70	57057	5.55	14	41^f^
151944059	Ape4	99	54140	6.54	15	37
207342034	Gas1	45	59545	4.46	1	3
323305878	Rnq1	191	23864	5.71	5	20

a GI number.

b Standard protein names are indicated.

c MASCOT protein score.

d Number of unique peptides matched to mass peaks.

e Sequence coverage in percent.

f Percent of coverage without GFP sequence is indicated.

Amyloidogenic proteins may form polymers which are sensitive to SDS at room temperature, but are resistant to treatment with a milder detergent, sarkosyl. For example, Pub1 forms SDS-sensitive but sarkosyl-resistant aggregates in [*PSI* ^+^] cells [28]. Based on this we isolated DRAFs from cell lysates of [*PIN* ^+^]/Aβ-GFP and [*pin* ^−^]/GFP strains using 3% sarkosyl. Subsequent 2D-DIGE analysis allowed detection of Aβ-GFP, as well as Rnq1, thus showing that both proteins form sarkosyl-insoluble aggregates (Fig. 2B, Table 2 and S2 Table). Notably, in contrast to SDS, sarkosyl treatment resulted in detection of numerous yellow spots which correspond to proteins present in both test and control samples (Fig. 2B). However, it is important that our comparative approach enabled easy discrimination between proteins which were present only in the test sample (red spots) and proteins forming detergent-resistant complexes present in both test and control samples (yellow spots). Thus, the numerous yellow spots would not interfere with the identification of a novel prion or amyloid protein if the control sample does not contain it. We identified all proteins corresponding to yellow spots in Fig. 2B. Of them, only Ape1, Ape4 and Gas1 derived from aggregates which were resistant to both sarkosyl and SDS. Surprisingly, most of the proteins forming sarkosyl-insoluble complexes were components of the 20S catalytic core of the proteasome. A list of all identified proteins is presented in Table 3 and S3 Table.

**Table 2:** Identification of proteins in sarkosyl-treated DRAFs isolated from the BY4742 [*PIN* ^+^] strain expressing Aβ-GFP or PrP-GFP.

Accession^a^	Protein b	Score^c^	Molecularmass	Calculatedisoelectric point	Peptidecount^d^	Coverage^e^
253723204	Aβ-GFP	137	2176	5.21	1	70^f^
240104235	PrP-GFP	310	13201	6.32	4	52^f^
408368764	Rnq1	94	18836	5.47	10	50

a GI number.

b Standard protein names are indicated.

c MASCOT protein score.

d Number of unique peptides matched to mass peaks.

e Sequence coverage in percent.

f Percent of coverage without GFP sequence is indicated.

**Table 3:** Identification of proteins present in DRAFs isolated by treatment with sarkosyl from the BY4742 [*PIN* ^+^] strain and its [*pin* ^−^] derivative.

Accession^a^	Protein^b^	Score^c^	Molecularmass	Calculatedisoelectricpoint	Peptidecount^d^	Coverage^e^	Function^f^
6322746	Ape1	275	57057	5.55	21	47	Vacuolar aminopeptidase 1
323308744	Ape4	277	53064	6.32	20	44	Aspartyl aminopeptidase 4
207342034	Gas1	203	48341	4.37	4	13	1,3-beta-glucanosyltransferase GAS1
6320849	Pre1	145	22503	5.83	9	58	Proteasome subunit beta type-4
11514002	Pre2	194	23286	5.94	10	53	Proteasome subunit beta type-5
11513426	Pre3	251	21481	5.36	16	88	Proteasome subunit beta type-1
3114282	Pre4	92	25903	5.75	9	47	Proteasome subunit beta type-7
298508225	Pre6	151	25337	6.26	9	45	Proteasome subunit alpha type-4
3114281	Pre7	120	24836	6.25	7	42	Proteasome subunit beta type-6
6323547	Pre8	550	27145	5.52	15	57	Proteasome subunit alpha type-2
3114271	Pre9	467	27003	5.71	9	46	Proteasome subunit alpha type-3
298508226	Pup2	217	27525	4.82	13	58	Proteasome subunit alpha type-5
323309416	Pup3	87	21263	5.17	5	30	Proteasome subunit beta type-3
6321826	Put2	247	64395	6.54	17	34	Delta-1-pyrroline-5-carboxylatedehydrogenase, mitochondrial
6321427	Scl1	217	27983	5.90	8	30	Proteasome subunit alpha type-1

a GI number.

b Standard abbreviations of protein names are indicated.

c MASCOT protein score.

d Number of unique peptides matched to mass peaks.

e Sequence coverage in percent.

f Data are presented in “Saccharomyces Genome Database” (http://www.yeastgenome.org/).

**Figure 3:**
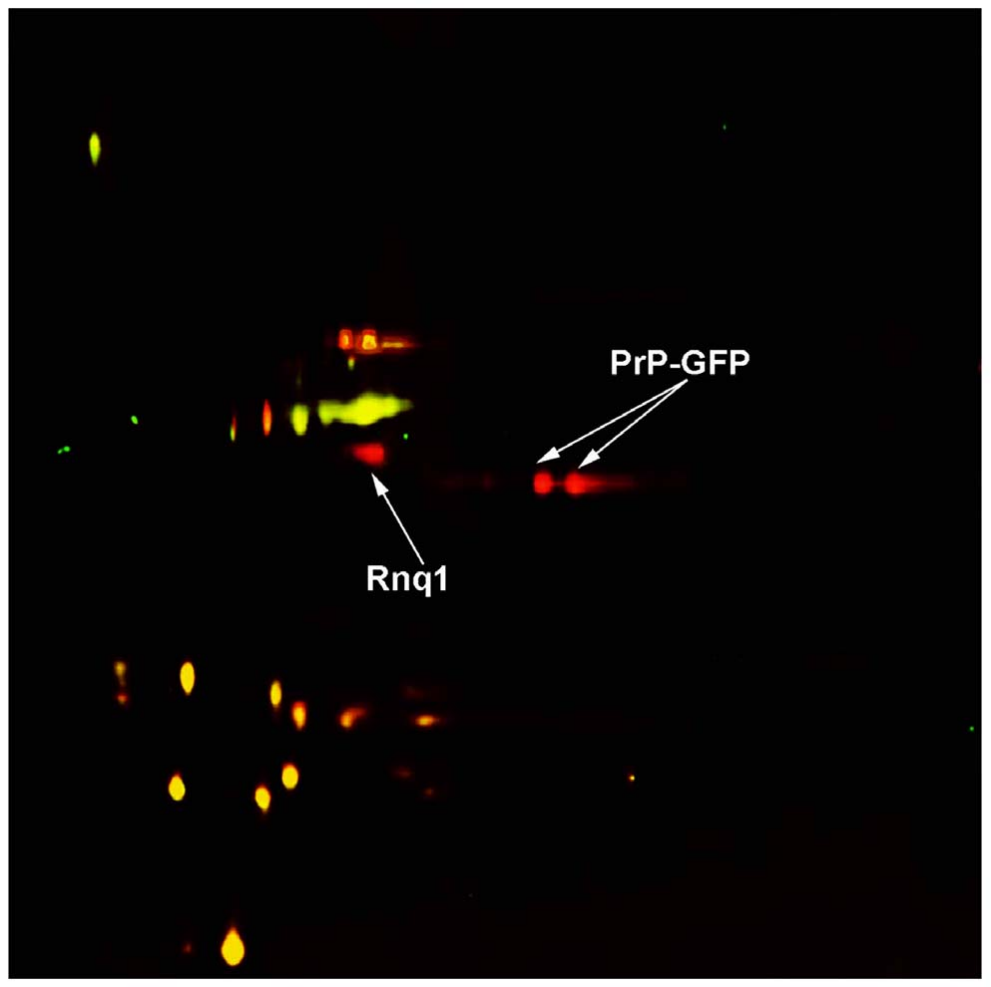
2D-DIGE image of proteins of sarkosyl-insoluble aggregates isolated from the BY4742 [*PIN* ^+^] strain expressing PrP-GFP and from its [*pin* ^−^] derivative expressing non-fused GFP. Spots corresponding to proteins from [*PIN* ^+^] cells expressing PrP-GFP are red, while those from GFP-containing cells (control) are green. Yellow spots correspond to proteins present in both compared samples. Identification data are presented in Table 2; mass spectra are shown in S2 Table.

Similar to Aβ-GFP, mouse PrP (90–231 aa) and its C-terminal fusion with GFP (PrP-GFP) form amyloid-like polymers in yeast cells independently of [*PIN* ^+^] [21], [25]. This allowed us to test whether PSIA enables detection of the amyloid form of PrP-GFP. As for cells expressing Aβ-GFP, the DRAFs were isolated from the strain BY4742 [*PIN* ^+^] expressing PrP-GFP and its [*pin* ^−^] variant expressing non-fused GFP using either 1% SDS or 3% sarkosyl. The red spots corresponding to the Rnq1 and PrP-GFP proteins were detected only in sample from the [*PIN* ^+^]/PrP-GFP strain treated with sarkosyl (Fig. 3, Table 2 and S2 Table), whereas, as was also shown above for Aβ-GFP, SDS-treated DRAFs isolated from this strain did not contain PrP-GFP (data not shown). This shows that sarkosyl allows identification of a wider range of amyloids than SDS. Notably, the yellow spots in Fig. 3 have a similar distribution as in Fig. 2B.

**Figure 4:**
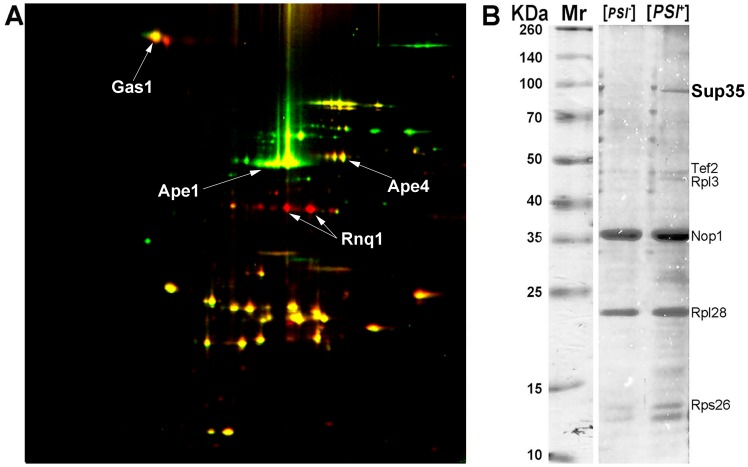
2D-DIGE image of proteins of sarkosyl-insoluble aggregates isolated from the GT81-1C [*PSI* ^+^][*PIN* ^+^] strain and its [*psi* ^−^][*pin* ^−^] derivative (A). Spots corresponding to proteins from [*PSI* ^+^][*PIN* ^+^] lysate and the [*psi^−^*][*pin* ^−^] control are red and green, respectively, while yellow spots correspond to proteins present in both compared samples. SDS-PAGE image of proteins from the same DRAFs that were insoluble in UTC buffer (B). Proteins identified by mass-spectrometry are indicated. Mr – protein molecular mass markers. Identification data are presented in Table 4; mass spectra are shown in S4 Table.

Next we tested whether PSIA allows detection of the Sup35 protein, which underlies another yeast prion determinant, [*PSI* ^+^]. For this we compared sarkosyl-insoluble fractions isolated from the GT81-1C [*PSI* ^+^][*PIN* ^+^] strain, which contains Sup35 and Rnq1 in prion form, and from its prion-free [*psi* ^−^][*pin* ^−^] derivative. Just like in previous experiment, we detected Rnq1 derived from lysate of the [*PSI* ^+^][*PIN* ^+^] strain (red spots in Fig. 4A). Surprisingly, no red spots corresponding to Sup35 were observed in the gel, suggesting that prion polymers of Sup35 were insoluble in UTC buffer used for 2D-DIGE. To check this possibility, the pellets insoluble in UTC were collected, solubilized by boiling in standard SDS-PAGE sample buffer with 2% SDS and analyzed by SDS-PAGE. Mass-spectrometric analysis of separated proteins revealed Sup35 in the [*PSI* ^+^][*PIN* ^+^] strain in significant amounts that could easily be detected by Coomassie Brilliant Blue staining (Fig. 4B, Table 4 and S4 Table). Thus, Sup35 forms amyloid polymers which are resistant not only to strong ionic detergents, but also to chaotropic agents present in UTC buffer, suggesting that analysis of aggregates insoluble in UTC buffer is a necessary step which allows identification of a wider range of amyloidogenic proteins. In contrast to Sup35, several other proteins were identified in lysates of both compared strains and, therefore might represent components of protein complexes constitutively present in yeast cells, which are insoluble in sarkosyl and UTC buffer.

**Table 4:** Identification of UTC-insoluble proteins in DRAFs isolated using sarkosyl from the GT81-1C [*PSI^+^*][*PIN* ^+^] strain and its [*psi^−^*][*pin* ^−^] derivative.

Accession a	Protein^b^	Score^c^	Molecularmass	Calculatedisoelectric point	Peptidecount^d^	Coverage^e^	Function^f^
6320190	Nop1	160	34444	10.24	15	36	Histone glutamine methyltransferase
323305009	Rpl28	130	13056	9.90	8	54	Ribosomal 60S subunit protein L28
49258841	Rpl3	88	43599	10.29	11	29	Ribosomal 60S subunit protein L3
6320978	Rps26	66	13438	10.90	6	33	Ribosomal 40S subunit protein S26
380005367	Sup35*	143	76535	6.11	15	25	Translation termination factor eRF3
32563240	Tef2	129	41199	8.36	13	38	Translation elongation factor 1-alpha

a GI number.

b Standard abbreviations of protein names are indicated.

c MASCOT protein score.

d Number of unique peptides matched to mass peaks.

e Sequence coverage in percent.

f Data are presented in “Saccharomyces Genome Database” (http://www.yeastgenome.org/).

*Detected only in the GT81-1C [*PSI^+^*][*PIN* ^+^] strain.

### Identification of proteins, whose polymerization is seeded by human huntingtin with an expanded polyQ tract

**Figure 5:**
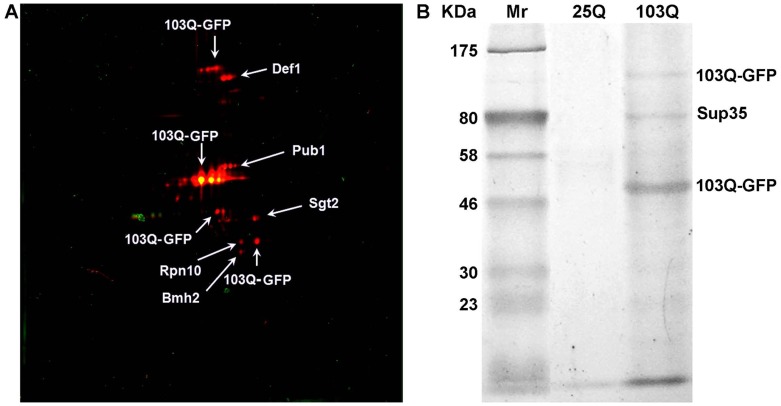
Identification of proteins forming SDS-insoluble aggregates in cells expressing 103Q-GFP. 2D-DIGE image of proteins of aggregates isolated using SDS (A). Spots corresponding to proteins from cells expressing 103Q-GFP and 25Q-GFP (control) are red and green, respectively. SDS-PAGE image of proteins from the same DRAFs that were insoluble in UTC buffer (B). Mr – protein molecular mass markers. Proteins identified by mass-spectrometry are indicated. Identification data are presented in Table 5 and S5 Table.

It is known that amyloid polymers of huntingtin can sequester cellular amyloidogenic proteins via induction of their polymerization. For instance, polymers of mutant human huntingtin formed in yeast cells were shown to seed polymerization of Sup35 and several other tested amyloidogenic proteins [28], [29]. Cross-seeded polymerization of host proteins was also observed in mammalian cells with polymers of elongated polyQ proteins [30]–[34]. Here, we used the PSIA approach for an unbiased identification of yeast proteins forming detergent-insoluble amyloid-like aggregates in cells expressing mutant human huntingtin. For this, we compared the protein composition of detergent-insoluble aggregates purified from cells of the 74-D694 strain expressing aggregation-prone huntingtin with expanded polyQ (103Q) and from control cells expressing non-aggregating huntingtin with short polyQ (25Q), both C-terminally tagged by GFP [35]. To limit the number of non-amyloidogenic proteins in the DRAFs, we used SDS in purification procedures, though, as was shown above, this could result in underestimation of the number of aggregating proteins. Taking into consideration the observation that huntingtin aggregates are difficult to dissolve, we solubilized them by treatment with formic acid [36] before adding UTC buffer. Proteins incubated in UTC buffer were separated into soluble and insoluble fractions by centrifugation. Soluble proteins derived from lysates with 103Q-GFP and 25Q-GFP were stained with fluorescent dyes and analyzed by 2D-DIGE. The proteins identified in 103Q-GFP-expressing cells were 103Q-GFP, Pub1, Sgt2, Rpn10, Def1 and Bmh2 (Fig. 5A, Table 5 and S5 Table). Remarkably, like most yeast prion proteins, the identified proteins, except Sgt2, contain regions which are pronouncedly enriched in Q and/or N residues. It is interesting that 103Q-GFP was identified in spots of different mobility. The same has been observed for huntingtin isolated from mammalian cells [20], [37] most probably due to transglutaminase-mediated covalent cross-linking of huntingtin monomers. Importantly, transglutaminase activity has also been demonstrated in *S. cerevisiae* [38], [39]. Proteins insoluble in UTC buffer were separated by SDS-PAGE and identified by mass-spectrometry. Apart from 103Q-GFP, we detected Sup35 (Fig. 5B, Table 5 and S5 Table), as expected [28]. Thus, 103Q-GFP amyloid induces appearance of SDS-insoluble aggregates of at least six yeast proteins, five of which have obvious Q/N-rich domains.

**Table 5:** Identification of proteins that polymerize in the presence of huntingtin aggregates.

Accession^a^	Protein^b^	Score^c^	Molecularmass	Calculatedisoelectricpoint	Peptidecount^d^	Coverage^e^	Function^f^	Proteinabundance(molecules per cell)^g^
398365597	Bmh2	119	31042	4.82	7	28	14-3-3 protein	∼47600
6322796	Def1	106	83923	4.92	9	17	RNAPII degradation factor	∼3400
6324312	Pub1	78	50731	4.95	6	14	Poly (A)+RNA-binding protein	∼49600
398365027	Rpn10	120	29729	4.73	6	26	Proteasome component	∼17200
6324580	Sgt2	133	37195	4.68	11	39	Glutamine-richcytoplasmic co-chaperone	∼9420^h^
380005303	Sup35	79	76503	6.54	8	29	Translationtermination factor eRF3	∼79000

a GI number.

b Standard protein names are indicated.

c MASCOT protein score.

d Number of unique peptides matched to mass peaks.

e Sequence coverage in percent.

f Data is presented in “Saccharomyces Genome Database” (http://www.yeastgenome.org/).

g Protein abundance data is taken from [27].

h Sgt2 levels are have been shown to increase in response to the presence of amyloids [47].

## Discussion

The PSIA approach developed in this work allowed us to detect all the tested proteins with well established capability for amyloid formation, such as yeast Rnq1 and Sup35 prion proteins as well as mammalian PrP, Aβ and mutant huntingtin tagged with GFP. Notably, the obtained results depended on the detergent used for amyloid isolation. The use of either SDS or sarkosyl allowed detection of Rnq1, Sup35 and Q103-GFP, while PrP-GFP and Aβ-GFP were detected only with the use of sarkosyl, because their aggregates are not resistant to SDS treatment. Solubility in 1% SDS was earlier shown for Aβ aggregates formed in mammals [24]. Thus, the use of sarkosyl for aggregate isolation enables identification of amyloid proteins which cannot be isolated with the use of SDS. It is also worth to stress that our results provide new insight into resistance of amyloids to detergents and solvents (Table 6). The only characteristic shared by all tested amyloids, is the resistance to treatment with 3% sarkosyl at room temperature. Also, some amyloids, such as prion polymers of Sup35, are insoluble in UTC buffer used for 2D-DIGE, which contains chaotropic agents, and even in formic acid, but can be efficiently dissolved by boiling in the presence of 2% SDS. This complicates the method, since solubilization of various amyloids will requires different solvents. Another limitation of PSIA is that extremely acidic and basic proteins cannot be detected by 2D-DIGE. Thus, a small portion of UTC-soluble proteins is excluded from the analysis.

**Table 6:** Resistance of amyloids to solubilizing agents.

Amyloids	Resistance to treatment with:				
	Sarkosyl, 3%	SDS, 1%	UTC (Urea, 8 M, thiourea,2 M, CHAPS, 4%)	SDS, 2%,boiling	Formic acid,95%
Rnq1	+	+	−	−	N/D
Sup35	+	+	+	−	+
PrP-GFP	+	−	−	−	N/D
Aβ-GFP	+	−	−	−	N/D
103Q-GFP	+	+	+	+	−

“+”, resistant.

“−”, sensitive.

All types of treatment were carried out at room temperature, unless noted otherwise.

Remarkably, color selection provided by the 2D-DIGE procedure in PSIA makes this approach especially convenient for the identification of proteins derived from aggregates which differ in compared samples, such as prion proteins or proteins of amyloids whose appearance is related to pathology. Indeed, besides proteins detected to validate this approach, six huntingtin-associated proteins were also identified in this way, for two of which, Pub1 and Def1, the ability to form amyloid fibrils was shown earlier [9].

It should be noted that the use of sarkosyl in PSIA is not well suited for the identification of constitutive amyloids that may be present in both test and control samples. This is due to the fact that sarkosyl treatment does not solubilize some protein complexes of non-amyloid nature. Most of the presumed non-amyloid proteins represent proteasomal components, which indicates resistance of 20S catalytic core of yeast proteasome to 3% sarkosyl rather than their amyloid origin. Earlier, the proteasome was shown to be resistant to some non-ionic detergents, such as Triton X-100 [40]. However, other proteins, especially those which were derived from the most stable aggregates resistant to such a strong detergent as SDS, e.g. Gas1, Ape1 and Ape4, may represent proteins of constitutive amyloids. Of course, in all cases, a set of additional experimental assays is necessary to verify whether a new protein candidate identified by PSIA is indeed amyloidogenic or behaves like a prion.

The developed approach also allowed us to extend the work on characterization of the ability of amyloids to induce polymerization of endogenous yeast proteins. Earlier it was demonstrated that polymers of proteins with extended polyQ domains, including mutant human huntingtin, caused appearance of SDS-insoluble aggregates of some chromosomally-encoded Q/N-rich proteins [28], [41], [42]. Here, we show that amyloids of mutant human huntingtin induce appearance of SDS-insoluble aggregates of at least six host proteins, Def1, Pub1, Rpn10, Bmh2, Sgt2, and Sup35. Notably, with one exception, all these proteins contain Q- or Q/N-rich tracts of different lengths supporting our suggestion that such proteins can interdependently form amyloids [28]. It may however seem surprising that among the multitude of yeast Q/N-enriched proteins only these formed detectable aggregates in response to appearance of amyloids of mutant huntingtin. It is clear that the capability of a protein to aggregate should depend on its expression level, however with the exception of Sup35, identified proteins are only modestly expressed (Table 5). Nevertheless, proteins enriched with Q and N may differ from each other by their intrinsic propensity to polymerize and the detected proteins may be among those which are most prone to polymerization. Except for Sup35, whose polymerization substantially contributes to toxicity of mutant huntingtin in yeast [41]–[43], other identified proteins are non-essential and therefore their polymerization-mediated inactivation modulates rather than causes a cytotoxic effect. In support of this, deletion of the *DEF1* gene alleviated huntingtin toxicity in yeast [44], while Def1 was shown to colocalize with huntingtin aggregates [45]. Sgt2 was also detected in huntingtin inclusions [46] and has been proposed to be an amyloid sensor [47]. Rpn10 and Bmh2 have not been previously shown to interact with huntingtin polymers, however Bmh1, which is highly similar to Bmh2, has been detected in huntingtin aggregates [46] and shown to play a role in huntingtin toxicity via aggresome formation [48]. The finding that many functionally unrelated proteins form polymers in response to the appearance of huntingtin amyloids may explain the diversity of lesions typical for Huntington pathology, especially keeping in mind that such polymers can sequester other proteins, which interact with these polymers, as was earlier shown in a yeast model for the Sup45 protein which binds to Sup35 amyloid polymers [42], [43], [49].

Another recently published approach to the identification of amyloid proteins, called TAPI (Technique for Amyloid Purification and Identification) is based on inability of SDS-resistant aggregates to migrate into polyacrylamide gel, if boiling of the sample is omitted [23]. However, it seems that the selectivity of TAPI is not sufficiently high and, therefore, proteins which, in all probability, do not form detergent-resistant complexes are also trapped at the top of the gel. In favor of this, non-overlapping sets of non-prion proteins were identified in [*PSI* ^+^][*PIN* ^+^] and [*psi* ^−^][*pin* ^−^] cells. Remarkably, TAPI and PSIA resulted in identification of different huntingtin-associated yeast proteins, among which only one protein, Pub1, was identified in both screens. However, most of the proteins revealed by PSIA in cells expressing mutant huntingtin contain Q/N-rich domains and were included in the list of potential prion proteins [50], while only two, Pub1 and Ynl208w, of those identified by TAPI are of this class. At last, contrary to PSIA, TAPI did not identify Sup35 among proteins whose polymerization is cross-seeded by mutant huntingtin, despite it being a major source of its toxicity in yeast [41]–[43].

To conclude, we would like to stress that the validation of the approach developed in this work demonstrated its efficacy for the detection of all tested proteins with known capability for amyloid formation and allowed identification of novel proteins, whose polymerization is cross-seeded by mutant human huntingtin. Taken together, the results of this work demonstrate that PSIA may not only significantly facilitate identification of amyloid proteins in entire proteomes, which is necessary for further clarification of the role of amyloids in the regulation of cellular processes, pathogenesis and aging, but can also provide an efficient instrument for revealing new prion proteins.

## Materials and Methods

### Strains, growth conditions and plasmids

The yeast strains along with their genotypes are listed below: GT81-1C, *MATa ade1–14 his3-Δ200 leu2–3,112 lys2–801 trp1–289 ura3–52* [*PSI^+^*][*PIN^+^*] [51]; GT159, *MATa ade1-14 his3 leu2 lys2 trp1-*Δ *ura3* [*psi^−^*][*PIN^+^*] [52]; BY4742, *MATα his3*-Δ*1 leu2*-Δ*0 lys2*-Δ*0 ura3*-Δ*0* [*psi^−^*][*PIN^+^*] (Invitrogen) and their [*psi^−^*][*pin^−^*] derivatives obtained by curing of [*PIN^+^*] and [*PSI^+^*] by guanidine hydrochloride treatment; 74-D694, *MATa ade1-14 his3*-Δ*200 trp1-289 ura3-52 leu2-3,112* [*psi^−^*][*PIN^+^*] [53]. Yeast cultures were grown at 30°C in the liquid or solid complete (YPD, 1% yeast extract, 2% peptone, 2% glucose) or synthetic (SC, 0.67% yeast nitrogen base, 2% glucose supplemented with the required amino acids) media. The *GAL1/10* promoter was induced as described [42].

The multicopy *URA3* plasmids pGPD-PrP(90–231)-GFP [54] and pU-Aβ-GFP [25] express under the control of *GPD* promoter the fragment of hamster PrP (90–231 aa) and the human beta-amyloid peptide (1–40 aa), respectively, both C-terminally fused to GFP (Green Fluorescent Protein). The multicopy *URA3* 103Q-GFP and 25Q-GFP plasmids [35] encode the first exon of the human huntingtin gene with either 103 or 25 codons for glutamine, fused in frame to the GFP-encoding sequence. Expression of the 103Q-GFP- and 25Q-GFP-encoding genes from these plasmids is under the control of the inducible *GAL1/10* promoter. DNA transformation of lithium acetate-treated yeast was done as described [55].

### Isolation of detergent-resistant aggregate fractions

A detailed protocol of the whole procedure is shown in the right panel of Fig. 1. Yeast cells were lysed in Tris-buffered saline (TBS) (30 mM Tris-HCl, pH 7.4, 150 mM NaCl), supplemented with 10 mM PMSF and Complete Protease Inhibitor (Roche) by beating with glass beads. The obtained lysates were precleared (2500 g, 10 min, 4°C) and then fractionated by ultracentrifugation (151000 g, 2 h, 4°C). Pellets containing protein polymers were resuspended in TBS with 200 µg/ml RNAse A, incubated for 15 min and treated with 3% sarkosyl for 5 min, all at room temperature. In some cases, as described in Results, 3% sarkosyl was substituted with 1% SDS. Then, detergent-resistant protein complexes were separated from monomeric proteins by ultracentrifugation at 151000 g, 8 h, 4°C (16°C in the case of SDS treatment) through 25% sucrose-TBS cushion with 0.3% sarkosyl (or 0.1% SDS, if SDS was used for treatment). Pellets were suspended in TBS with 0.05% Tween-20 and sedimentated again for at 151000 g (2 h, 4°C) to remove ionic detergents. The described procedure is based on [22], with modifications that include the use of sarkosyl instead of SDS as well as the omission of one centrifugation step in the presence of detergent and addition of a centrifugation step in order to remove ionic detergents from the final pellets.

### Solubilization and labeling of proteins

Pellets (10 µl) were dissolved in UTC buffer (8 M urea, 2 M thiourea, 4% CHAPS, and 30 mM TrisHCl pH 8.5) for 2 h at room temperature (Fig. 1, left panel) and analyzed by 2D-DIGE. Proteins insoluble in UTC buffer were collected by centrifugation (12000 g, 15 min, room temperature) and used for further analysis. For the analysis of huntingtin aggregates, prior to resuspension in UTC buffer, the pellets were dehydrated by washing with acetone 3 times (pellet was resuspended in 1 ml acetone, centrifuged for 2 min at 13400 g and supernatant was removed), air dried for 30 min and then dissolved in 300–1000 ml of formic acid, depending on the size of the initial pellet in order to disaggregate huntingtin [36]. Resuspension was aided by light ultrasonication (30% amplitude, 2 sec pulse, 3 sec rest for 30 sec, 3 mm tip) on a VCX130 ultrasonic homogenizer (Sonics, USA). The formic acid was then removed using a vacuum rotary evaporator. The proteins soluble in UTC buffer from test and control samples were labeled at lysine residues with Cy5 and Cy3 (CyDye DIGE Fluor, N-hydroxysuccinimide-activated esters, BioDye, Russia), correspondingly, according to recommendations of the manufacturer. The quantity of labeled proteins was estimated by SDS-PAGE.

### Separation of proteins

The proteins soluble in UTC buffer were separated by 2D-DIGE [26]. For this, isoelectrofocusing was performed using glass capillaries (18 mm) in 4% PAAG (2.4% CHAPS/NP40, 8 M urea, 2% ampholines 3–10 (BioRad)). Then, tube gels were ejected, incubated (10 min) in equilibration buffer (40% (w/v) glycerol, 125 mM TrisHCl, 3% (w/v) SDS, 65 mM DTT (pH 6.8)), and separated by SDS-PAGE in a gradient gel (8–16%). A laser fluorescence scanner (Fuji) was used to detect the dye fluorescence. Excitation and emission wavelengths for Cy3 and Cy5 were 532/580, and 635/670 nm, respectively, which are not affected by GFP fluorescence (488/509 nm) of the fusion proteins used in this study. After that the gels were re-stained with silver nitrate [56], and protein spots of interest were excised and saved for mass-spectrometric analysis.

The proteins insoluble in UTC buffer were boiled in standard SDS-PAGE sample buffer, separated by SDS-PAGE (12% gel) and stained with Coomassie Brilliant Blue R-250 (BioRad). The protein bands from the gels were excised and subjected to the same procedure for mass-spectrometry as in the case of spots from 2D-gels.

### Identification of proteins

Gel slices were washed twice with deionized water and washed once with 40% acetonitrile in 50 mM ammoniumbicarbonate solution. Next, dehydration was performed in 100% acetonitrile followed by removing of liquid and air-drying of gel slices. The dried samples were incubated for 4 h with 5 µl of sequencing grade trypsin (Promega) 5 µg/ml solution, 100 mM ammonium bicarbonate (pH 7.0) at 37°C. Peptides were extracted with 5 µl of 0.5% trifluoroacetic acid in water. Mass spectrometric peptide analysis was performed using an Ultraflextreme MALDI-TOF/TOF mass spectrometer (Bruker Daltonics, DE) equipped with an Nd laser (354 nm) in reflecto-mode (the mass range 700–4500 m/z). The matrix was α-cyano-4-hydroxycinnamic acid. Peak lists were generated by the flexAnalysis 3.2 software (Bruker Daltonics). Proteins were identified by Mascot software release version 2.4.2 (Matrix Science, http://www.matrixscience.com) in the database of National Center for Biotechnology Information (NCBI). Mass tolerances were set to default values. Modifications (propionamidomethylation of cysteine residues and partial oxidation of methionine) were permitted for peptide mass fingerprint searches. One missed cleavage was allowed. Identification data were compared with the location of the corresponding proteins in the gel.

## Supporting Information

S1 Table Identification data of the proteins listed in Table 1.
(PDF)

S2 Table Identification data of the proteins listed in Table 2.
(PDF)

S3 Table Identification data of the proteins listed in Table 3.
(PDF)

S4 Table Identification data of the proteins listed in Table 4.
(PDF)

S5 Table Identification data of the proteins listed in Table 5.
(PDF)
