# Supplementary material for: Proteomic Screening for Amyloid Proteins
Source: PLoS One. 2014 Dec 30;9(12):e116003. doi: 10.1371/journal.pone.0116003 (PMC4280166; doi:10.1371/journal.pone.0116003)
Supplement: S1 Table — Identification data of the proteins listed in Table 1. (PDF) [file pone.0116003.s001.pdf]

Table S1. Identification data of the proteins listed in Table 1.

Ape1

Mass-spectrum:

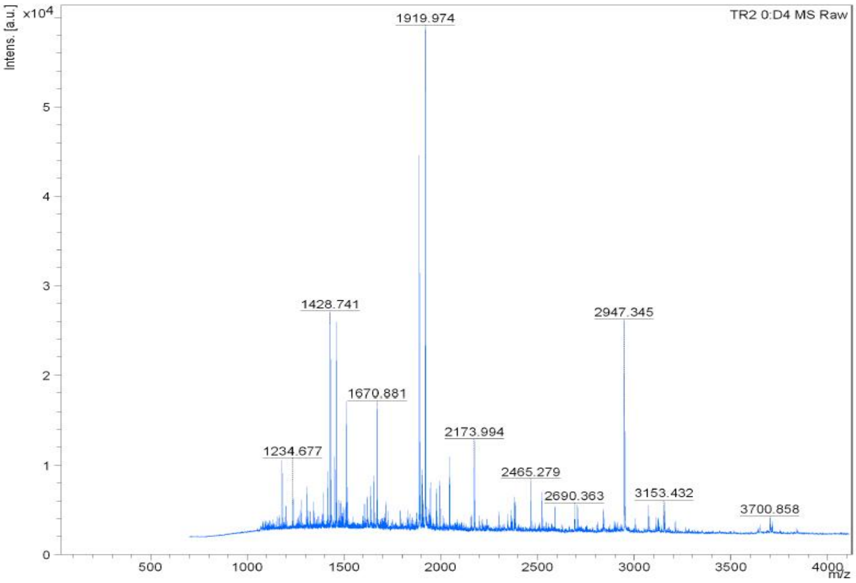

Identification:

gi|6322746 Mass: 57057 Score: 70 Expect: 0.00084 Matches: 14

Start–EndObservedMr(expt)Mr(calc)ppm MPeptide 34–623647.61403646.60683646.6925–23.52K.EKKENENSWCILEHNYEDIAQEFIDFIYK.N 89–1011392.59931391.59211391.5953–2.360K.SNWQDSIGEDGGK.F 107–1181234.67701233.66971233.6717–1.610R.NGTNLSAFILGK.N 125–1391451.81291450.80561450.8144–6.050K.GVGVIGSHVDALTVK.L 155–1711887.98201886.97481886.9890–7.570R.IAVAPYGGTLNELWLDR.D 191–2031414.72431413.71701413.7286–8.190K.SALVDSTPLPVCR.I + Carbamidomethyl (C) 204–2211907.97741906.97011906.9941–12.60R.IPSLAPHFGKPAEGPFDK.E 266–2862362.19712361.18982361.2138–10.10K.LAGVEVSELIQMDLDLFDVQK.G 266–2862378.19992377.19262377.2087–6.770K.LAGVEVSELIQMDLDLFDVQK.G + Oxidation (M)

Start–EndObservedMr(expt)Mr(calc)ppm MPeptide 302–3202173.99442172.98712173.0733–39.71R.LDDRLCSFAAMIALICYAK.D + Carbamidomethyl (C) 321–3462947.34472946.33752946.3618–8.260K.DVNTEESDLFSTVTLYDNEEIGSLTR.Q 452–4671459.73751458.73021458.7427–8.530R.SGGTIGPSLASQTGAR.T 468–4821654.87931653.87201653.8872–9.190R.TIDLGIAQLSMHSIR.A 468–4821670.88101669.87371669.8821–5.040R.TIDLGIAQLSMHSIR.A + Oxidation (M)

No match to: 1080.4900, 1093.5298, 1107.5346, 1134.6055, 1148.6715, 1157.5842, 1194.5870, 1201.6817, 1235.5134, 1235.6625, 1265.6320, 1273.6445, 1302.6991, 1311.6604, 1323.6909, 1340.6796, 1346.3023, 1365.6458, 1428.7412, 1434.7418, 1479.7549, 1485.7632, 1489.7369, 1491.7273, 1493.7094, 1496.7688, 1507.7355, 1510.7271, 1516.7542, 1595.8165, 1606.8789, 1618.2236, 1636.6241, 1707.7649, 1727.8935, 1826.8836, 1837.9352, 1851.9083, 1874.9528, 1891.9788, 1902.9665, 1904.9877, 1919.9737, 1935.9741, 1945.0007, 1976.9935, 2013.8892, 2045.8709, 2156.9911, 2195.9852, 2239.1189, 2299.8872, 2346.1122, 2522.3036, 2588.3215, 2690.3629, 2808.2913, 2840.2859, 2902.3547, 3004.3641, 3073.2506, 3112.5430, 3122.4456, 3153.4316, 3156.4217, 3210.4479, 3700.8584, 3710.6574

Ape4

Mass-spectrum:

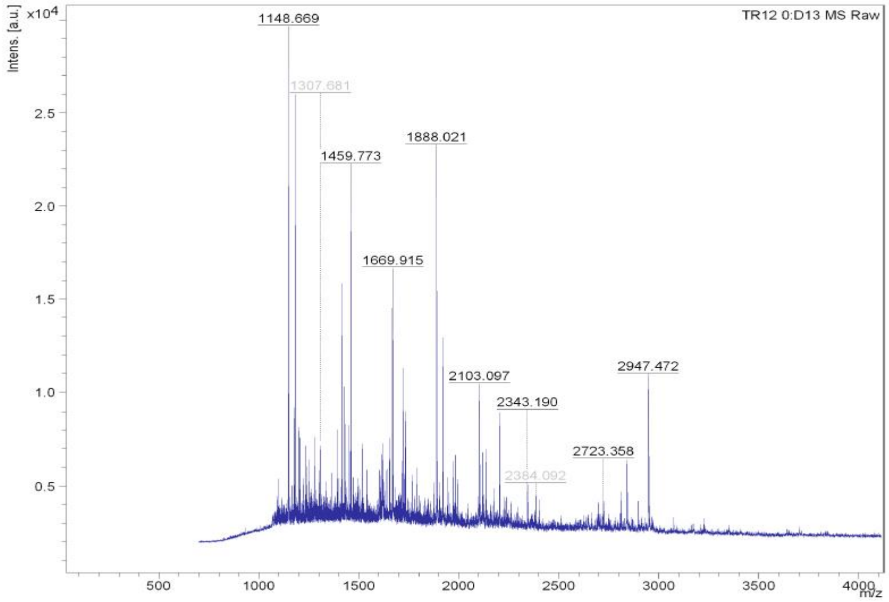

Identification:

gi|151944059 Mass: 54140 Score: 99 Expect: 1e-006
Matches: 15

Start-EndObservedMr(expt)Mr(calc)ppm MPeptide **21-**
402343.19032342.18312342.144416.50K.EFVSFLNSSHSPYHTVHNIK.K 55-
641098.53531097.52801097.52542.400R.DSWAGHVAQK.G 85-
1042103.09652102.08922102.054516.50K.WEPGNPIAITGAHTDSPALR.I 157-
1661180.73261179.72541179.7339-7.240R.LVDLNRPLLK.I 167-
1761148.66941147.66211147.6713-8.040K.IPTLAIHLDR.D 167-
1811733.00091731.99371731.963217.61K.IPTLAIHLDRDVNQK.F 187-
2001540.85491539.84761539.814421.50R.ETQLLPIGGLQEDK.T

Start-EndObservedMr(expt)Mr(calc)ppm MPeptide **187-**
2041970.12551969.11821969.036841.41R.ETQLLPIGGLQEDKTEAK.T 208-
2181251.62211250.61481250.61430.420K.EINNGEFTSIK.T 224-
2341201.69251200.68521200.6979-10.60R.HHAELLGLIAK.E 335-
3552202.19792201.19062201.096442.80K.GDGSDQTKPLFHSAILETSAK.S 375-
3901722.99441721.98711721.946423.60K.YESQHKPLLGGGPVIK.I 397-
4081337.72351336.71621336.70617.590R.YMTNSPGLVLVK.R + Oxidation (M) **460-**
4721394.69701393.68971393.672512.40R.ETGGSADLEFQIK.L 473-
4801115.58151114.57421114.5811-6.201K.LFKEFFER.Y

No match to: 1093.5318, 1102.5721, 1157.5937, 1174.6564, 1205.6884, 1221.6514, 1233.6414, 1235.6669, 1237.7508, 1263.6802, 1284.6829, 1365.6711, 1392.6561, 1414.7490, 1428.7730, 1434.7555, 1451.8451, 1459.7732, 1471.7849, 1486.8021, 1492.7765, 1496.7841, 1507.7814, 1516.8057, 1604.9002, 1606.8982, 1617.1755, 1618.2755, 1639.9103, 1653.9077, 1669.9151, 1671.9310, 1684.8978, 1727.9318, 1765.8105, 1873.0239, 1888.0212, 1904.0162, 1920.0167, 1945.0438, 1983.1290, 2119.0979, 2135.1014, 2723.3577, 2808.4010, 2839.3768, 2896.4312, 2947.4721

Gas1

Mass-spectrum:

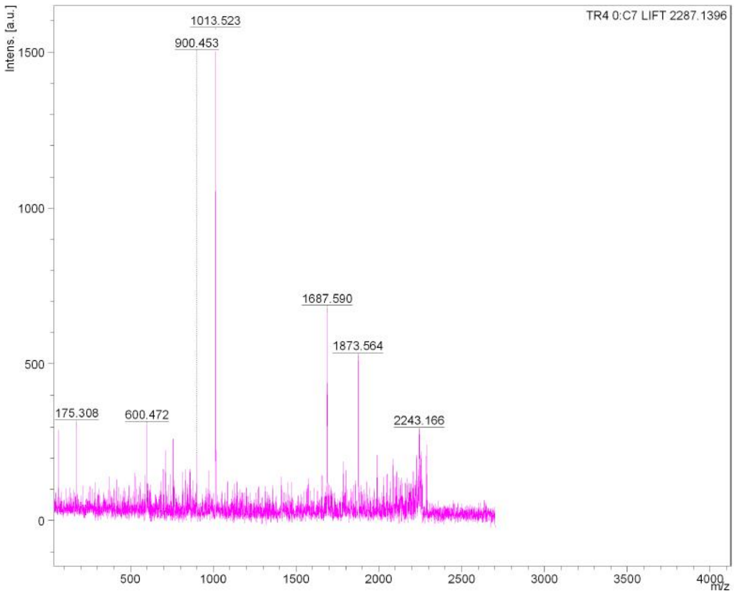

Identification (MS/MS):

gi|207342034 Mass: 59545 Score: 45 Match: 1

QueryStart-EndObservedMr(expt)Mr(calc)ppm MScoreExpectRank
1106-1272287.13962286.13232286.1855-
23.30450.000471UK.ALNDADIYVIADLAAPATSINR.

Rnq1

Mass-spectrum:

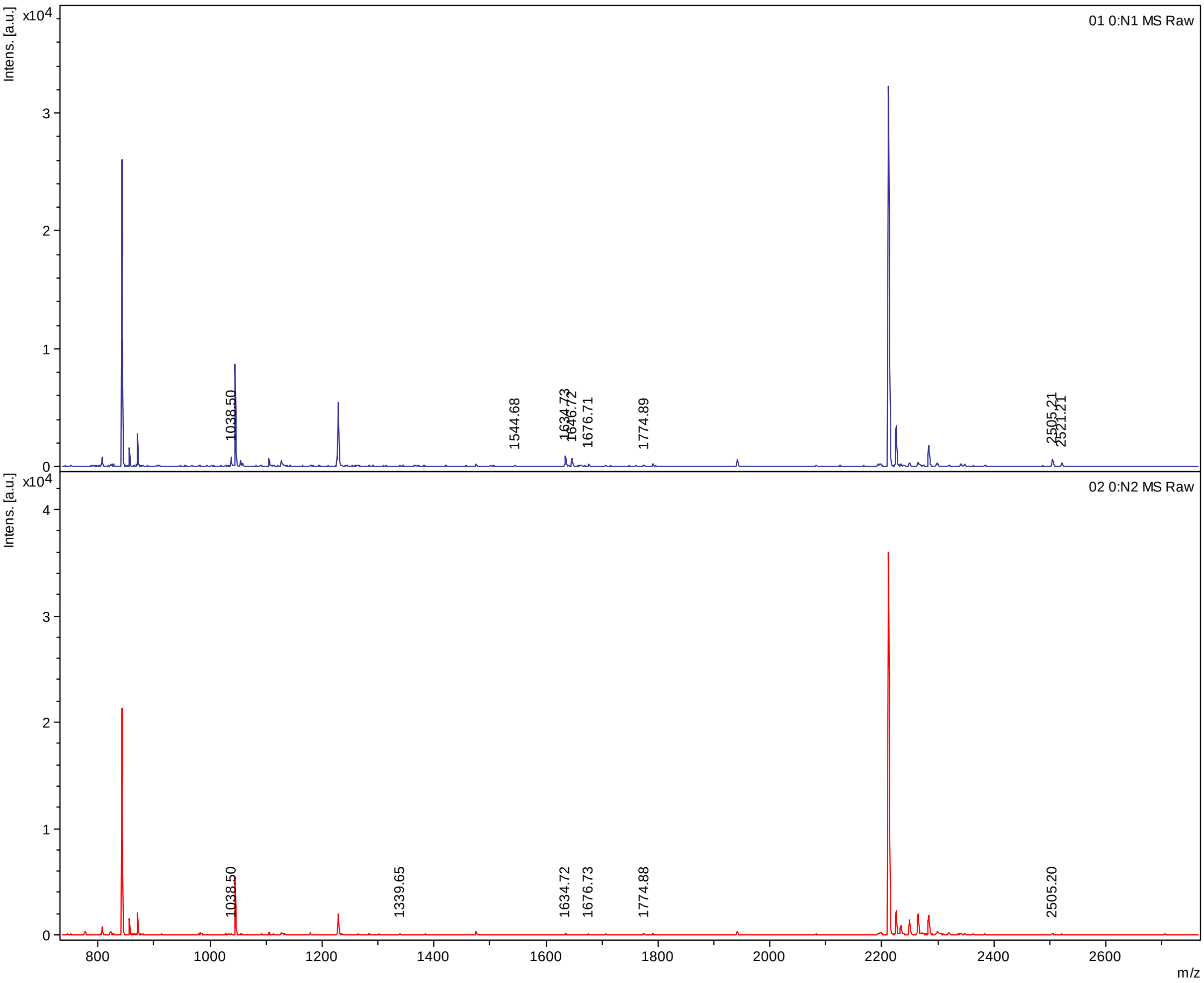

Identification:

gi|323305878 Mass: 23864 Score: 191 Expect: 1.4e-12 Matches: 5

Rnq1p [Saccharomyces cerevisiae FostersB]

Observed	Mr(expt)	Mr(calc)	ppm	Start	End	Miss	Ions	Peptide
1038.4986	1037.4913	1037.4964	-4.90	49 -	57	0	18	K.IAGYVMDNR.S
1660.7461	1659.7388	1659.7233	9.35	68 -	84	0	---	R.AAGGGSSFMNTLMADSK.G + Oxidation (M)
1676.7137	1675.7064	1675.7182	-7.02	68 -	84	0	---	R.AAGGGSSFMNTLMADSK.G + 2 Oxidation (M)
2505.2134	2504.2061	2504.2064	-0.12	26 -	48	0	136	K.LTSAAQSNPNDEQMSTIESLIQK.I
2521.2091	2520.2018	2520.2013	0.20	26 -	48	0	---	K.LTSAAQSNPNDEQMSTIESLIQK.I + Oxidation (M)

No match to: 1544.6808, 1633.7481, 1634.7260, 1645.7180, 1646.7205, 1774.8912
