# Supplementary material for: Proteomic Screening for Amyloid Proteins
Source: PLoS One. 2014 Dec 30;9(12):e116003. doi: 10.1371/journal.pone.0116003 (PMC4280166; doi:10.1371/journal.pone.0116003)
Supplement: S2 Table — Identification data of the proteins listed in Table 2. (PDF) [file pone.0116003.s002.pdf]

Table S2. Identification data of the proteins listed in Table 2.

Aβ-GFP

Mass-spectrum (MS/MS):

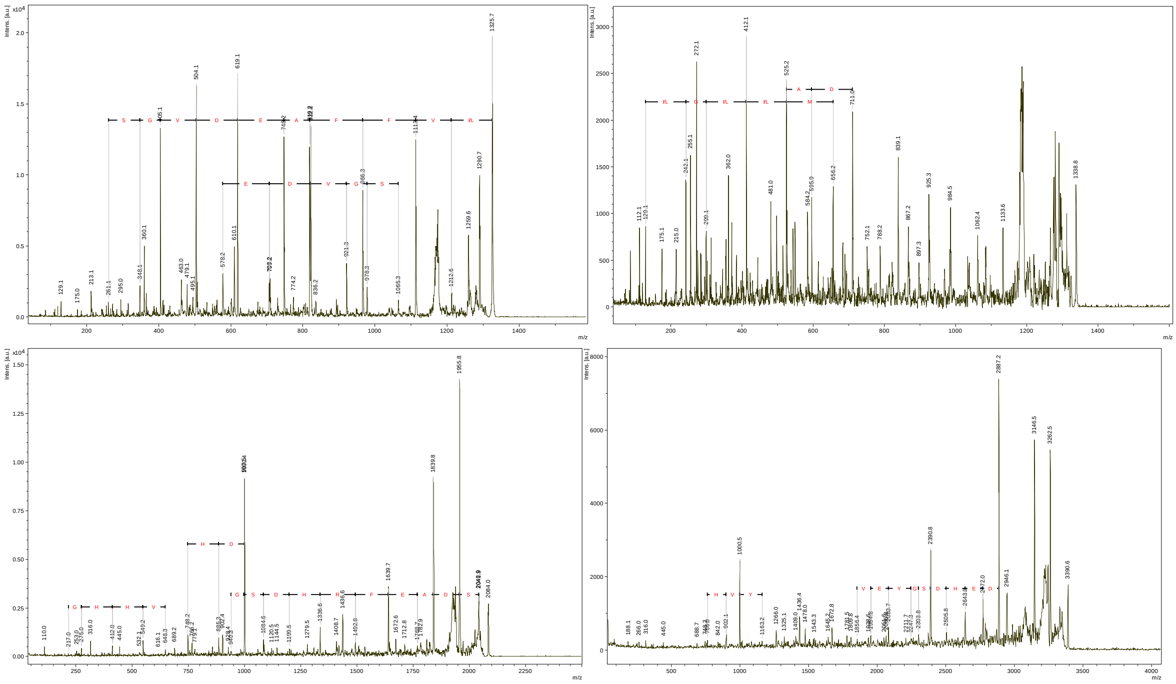

Identification:

gi|253723204 **Mass:** 2176 **Score:** **137** **Matches:** 1(1) **Sequences:** 1(1)

Chain A, Zinc-Binding Domain Of Alzheimer's Disease Amyloid Beta- Peptide In Water Solution At Ph 6.5

Query	Observed	Mr(expt)	Mr(calc)	ppm	Miss	Score	Expect	Rank	Unique	Peptide
19	2083.9300	2082.9227	2082.9144	3.99	1	143	9e-11	1	U	-.SDAEFRHDSGYEVHHQK.X + Acetyl (Protein N-term)

gi|177958 **Mass:** 3429 **Score:** **98** **Matches:** 1(1) **Sequences:** 1(1)

amyloid beta protein, partial [Homo sapiens]

Query	Observed	Mr(expt)	Mr(calc)	ppm	Miss	Score	Expect	Rank	Unique	Peptide
8	1325.6741	1324.6668	1324.6663	0.39	0	98	5.5e-06	1	U	K.LVFFAEDVGSNK.G

PrP-GFP

Mass-spectrum:

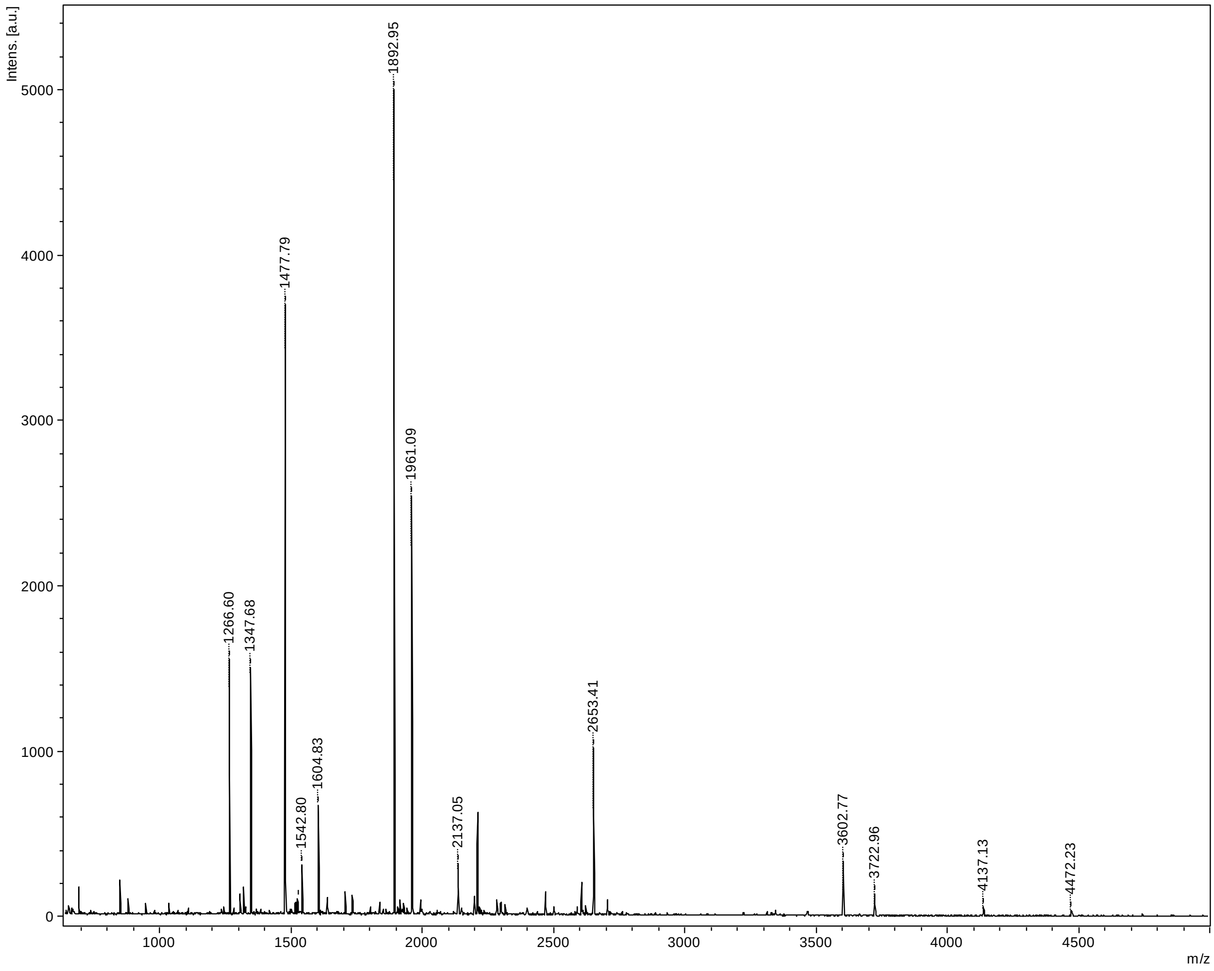

Identification:

gi|240104235 **Mass:** 13201 **Score:** **310** **Expect:** 2.7e-24 **Matches:** 4

Chain A, Mouse Prion Protein (121-231) With Mutations Y225a And Y226a

Observed	Mr(expt)	Mr(calc)	ppm	Start	End	Miss	Ions	Peptide
1526.7422	1525.7350	1525.7269	5.30	91 -	102	0	82	R.VVEQMCVTQYQK.E + Propionamide (C)
1542.7988	1541.7915	1541.7218	45.2	91 -	102	0	---	R.VVEQMCVTQYQK.E + Oxidation (M); Propionamide (C)
2137.0502	2136.0430	2136.0335	4.42	68 -	86	1	141	K.QHTVTTTTKGENFTETDVK.M
3602.7692	3601.7619	3601.7048	15.8	39 -	67	0	45	R.YPNQVYYRPVDQYSNQNNFVHDCVNITIK.Q + Propionamide (C)

No match to: 1266.5968, 1347.6805, 1477.7942, 1604.8267, 1892.9471, 1961.0884, 2653.4058, 3722.9558, 4137.1268, 4472.2296

Rnq1

Mass-spectrum:

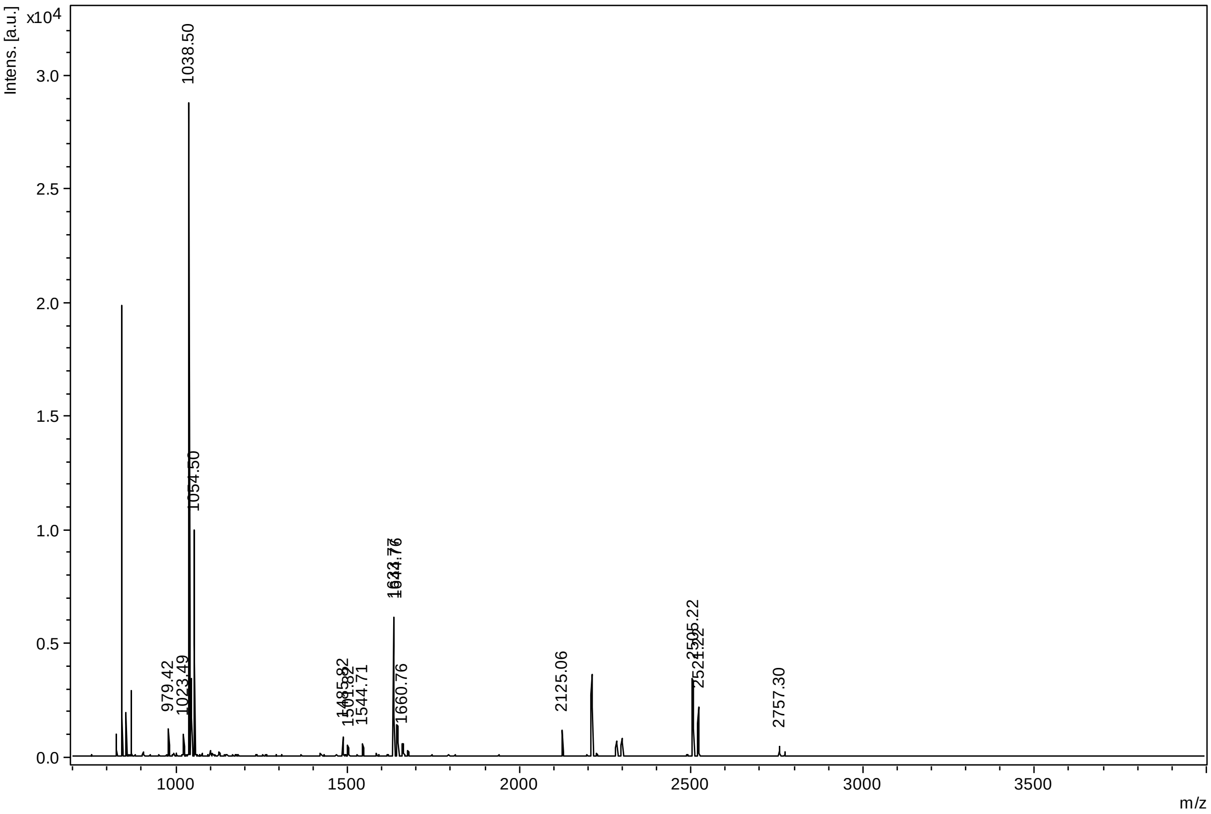

Identification:

gi|408368764 **Mass:** 18836 **Score:** **94** **Expect:** 0.0091 **Matches:** 10

truncated Rnq1p [Saccharomyces cerevisiae]

Observed	Mr(expt)	Mr(calc)	ppm	Start		End	Miss	Peptide
979.4196	978.4123	978.4003	12.3	58	-	67	0	R.SGGSDASQDR.A
1038.5017	1037.4944	1037.4964	-1.90	49	-	57	0	K.IAGYVMDNR.S
1054.5032	1053.4959	1053.4913	4.39	49	-	57	0	K.IAGYVMDNR.S + Oxidation (M)
1485.8229	1484.8156	1484.8021	9.11	94	-	107	0	K.LALLATVMTHSSNK.G
1501.8173	1500.8100	1500.7970	8.67	94	-	107	0	K.LALLATVMTHSSNK.G + Oxidation (M)
1644.7597	1643.7525	1643.7283	14.7	68	-	84	0	R.AAGGGSSFMNTLMADSK.G
1660.7558	1659.7485	1659.7233	15.2	68	-	84	0	R.AAGGGSSFMNTLMADSK.G + Oxidation (M)
2125.0618	2124.0546	2124.0236	14.6	6	-	25	0	K.LISEAESHFSQGNHAEAVAK.L
2505.2183	2504.2110	2504.2064	1.85	26	-	48	0	K.LTSAAQSNPNDEQMSTIESLIQK.I
2521.2183	2520.2111	2520.2013	3.86	26	-	48	0	K.LTSAAQSNPNDEQMSTIESLIQK.I + Oxidation (M)

No match to: 1023.4878, 1544.7086, 1633.7660, 2757.2960
