# Supplementary material for: Proteomic Screening for Amyloid Proteins
Source: PLoS One. 2014 Dec 30;9(12):e116003. doi: 10.1371/journal.pone.0116003 (PMC4280166; doi:10.1371/journal.pone.0116003)
Supplement: S3 Table — Identification data of the proteins listed in Table 3. (PDF) [file pone.0116003.s003.pdf]

Table S3. Identification data of the proteins listed in Table 3.

Ape1

Mass-spectrum:

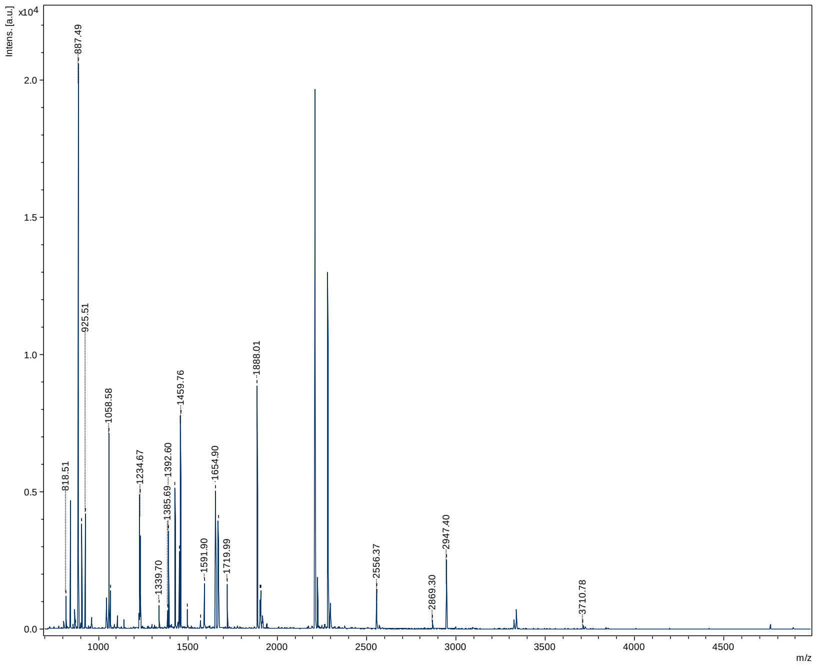

Identification:

gi|6322746 **Mass:** 57057 **Score:** 275 **Expect:** 5.7e-21 **Matches:** 21

Lap4p [Saccharomyces cerevisiae S288c]

Observed	Mr(expt)	Mr(calc)	ppm	Start	End	Miss	Peptide
818.5140	817.5068	817.5062	0.74	140 -	146	0	K.LKPVSFK.D
887.4889	886.4816	886.4814	0.31	295 -	301	0	K.HFLFAPR.L
906.4482	905.4410	905.4436	-2.91	496 -	502	0	K.FFNGFFK.H
925.5115	924.5042	924.5069	-2.90	441 -	447	0	K.VQYFQIK.N
1058.5822	1057.5750	1057.5768	-1.71	351 -	360	0	K.GGLLESVVER.S
1234.6698	1233.6626	1233.6717	-7.42	107 -	118	0	R.NGTNLSAFILGK.N
1339.6973	1338.6901	1338.6932	-2.33	437 -	447	1	R.NGDKVQYFQIK.N
1385.6914	1384.6841	1384.6829	0.85	496 -	505	1	K.FFNGFFKHWR.S
1392.6030	1391.5957	1391.5953	0.25	89 -	101	0	K.SNWQDSIGEDGGK.F
1428.7557	1427.7485	1427.7442	2.96	191 -	203	0	K.SALVDSTPLPVCR.I + Propionamide (C)
1451.8213	1450.8141	1450.8144	-0.24	125 -	139	0	K.GVGVIGSHVDALTVK.L
1459.7629	1458.7556	1458.7427	8.87	452 -	467	0	R.SGGTIGPSLASQTGAR.T
1570.8926	1569.8853	1569.8780	4.65	287 -	301	1	K.GTIGGIGKHFLFAPR.L
1591.8971	1590.8898	1590.8817	5.13	249 -	261	1	K.SPLFGKHCIHLLR.Y + Propionamide (C)
1654.9042	1653.8970	1653.8872	5.89	468 -	482	0	R.TIDLGIAQLSMHSIR.A
1670.8941	1669.8868	1669.8821	2.79	468 -	482	0	R.TIDLGIAQLSMHSIR.A + Oxidation (M)
1888.0082	1887.0009	1886.9890	6.28	155 -	171	0	R.IAVAPYGGTLNELWLDR.D
1908.0048	1906.9975	1906.9941	1.78	204 -	221	0	R.IPSLAPHFGKPAEGPFDK.E
2556.3653	2555.3580	2555.3496	3.30	155 -	178	1	R.IAVAPYGGTLNELWLDRDLGIGGR.L
2869.2972	2868.2899	2868.2825	2.58	222 -	247	0	K.EDQTIPVIGFPTPDEEGNEPPTDDEK.K
2947.3981	2946.3908	2946.3618	9.85	321 -	346	0	K.DVNTEESDLFSTVTLYDNEEIGSLTR.Q

No match to: 1066.5976, 1496.7924, 1719.9930, 1904.0052, 3710.7825

Ape4

Mass-spectrum:

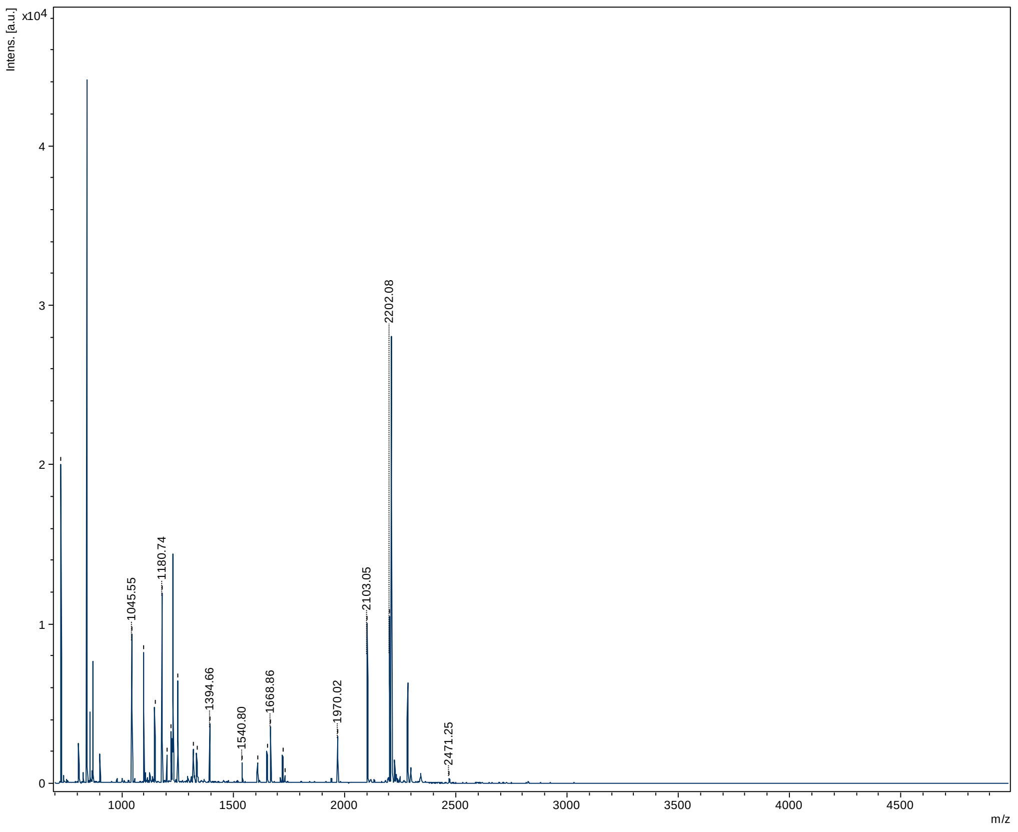

Identification:

gi|323308744 **Mass:** 53064 **Score:** 277 **Expect:** 3.6e-21 **Matches:** 20

YHR113W-like protein [Saccharomyces cerevisiae FostersO]

Observed	Mr(expt)	Mr(calc)	ppm	Start	End	Miss	Peptide
727.3534	726.3461	726.3337	17.1	468 -	472	0	K.EFFER.Y
1098.5154	1097.5081	1097.5254	-15.79	47 -	56	0	R.DSWAGHVAQK.G
1148.6728	1147.6655	1147.6713	-5.06	159 -	168	0	K.IPTLAIHLDR.D
1180.7408	1179.7335	1179.7339	-0.35	149 -	158	0	R.LVDLNRPLLK.I
1201.6905	1200.6832	1200.6979	-12.18	216 -	226	0	R.HHAELLGLIAK.E
1220.6387	1219.6314	1219.6561	-20.25	64 -	76	0	R.NGSSIIAFAVGGK.W
1251.6127	1250.6054	1250.6143	-7.05	200 -	210	0	K.EINNGEFTSIK.A
1321.7006	1320.6933	1320.7112	-13.49	389 -	400	0	R.YMTNSPGLVLVK.R
1337.6902	1336.6829	1336.7061	-17.33	389 -	400	0	R.YMTNSPGLVLVK.R + Oxidation (M)
1394.6616	1393.6543	1393.6725	-13.08	452 -	464	0	R.ETGGSADLEFQIK.L
1540.8023	1539.7950	1539.8144	-12.59	179 -	192	0	R.ETQLLPIGGLQEDK.T
1609.7817	1608.7744	1608.7995	-15.60	197 -	210	1	K.TEKEINNGEFTSIK.A
1652.8679	1651.8606	1651.8716	-6.62	437 -	451	0	R.TLDLGNPVLSMHSIR.E
1668.8605	1667.8533	1667.8665	-7.93	437 -	451	0	R.TLDLGNPVLSMHSIR.E + Oxidation (M)
1722.9360	1721.9287	1721.9464	-10.31	367 -	382	0	K.YESQHKPLLGGGPVIK.I
1732.9622	1731.9550	1731.9632	-4.73	159 -	173	1	K.IPTLAIHLDRDVNQK.F
1970.0244	1969.0171	1969.0368	-9.99	179 -	196	1	R.ETQLLPIGGLQEDKTEAK.T
2103.0450	2102.0378	2102.0545	-7.96	77 -	96	0	K.WEPGNPIAITGAHTDSPALR.I
2202.0842	2201.0769	2201.0964	-8.88	327 -	347	0	K.GDGSDQTKPLFHSAILETSAK.S
2471.2462	2470.2389	2470.2393	-0.16	13 -	33	1	K.EFVSFLNSSHSPYHTVHNIKK.H

No match to: 1045.5548

Gas1

Mass-spectrum:

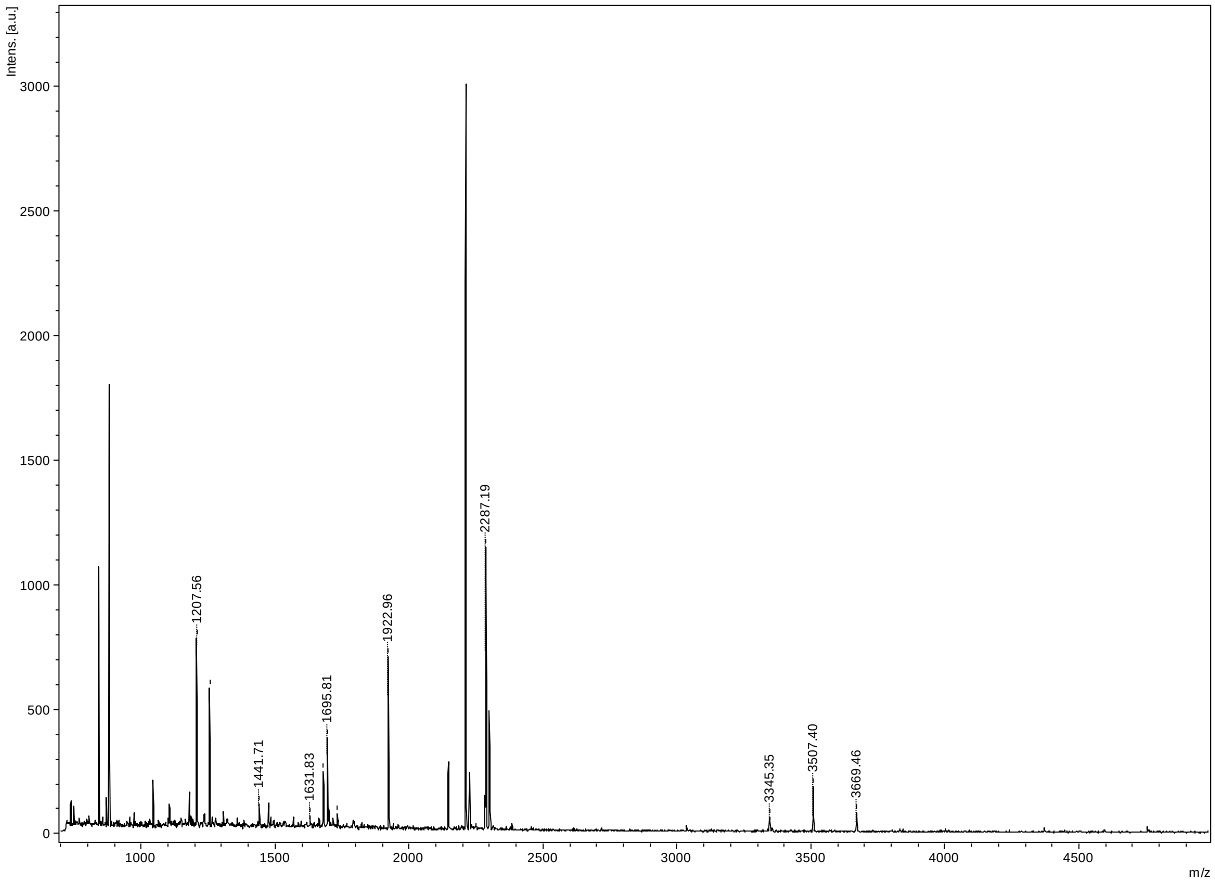

Identification:

gi|207342034 **Mass:** 48341 **Score:** 203 **Expect:** 1e-13 **Matches:** 4

YMR307Wp-like protein [Saccharomyces cerevisiae AWRI1631]

Observed	Mr(expt)	Mr(calc)	ppm	Start	End	Miss	Ions	Peptide
1207.5574	1206.5501	1206.5379	10.1	312 -	321	0	48	K.YGAYSFCTPK.E + Propionamide (C)
1679.8047	1678.7974	1678.7912	3.67	322 -	334	0	46	K.EQLSFVMNLYYEK.S + Oxidation (M)
1695.8069	1694.7996	1694.7748	14.6	90 -	104	1	---	R.KIPVGYSSNDDEDTR.V
2287.1896	2286.1824	2286.1855	-1.39	3 -	24	0	69	K.ALNDADIYVIADLAAPATSINR.D

No match to: 1255.6590, 1441.7122, 1631.8298, 1732.7874, 1922.9551, 3345.3503, 3507.3990, 3669.4629

Pre1

Mass-spectrum:

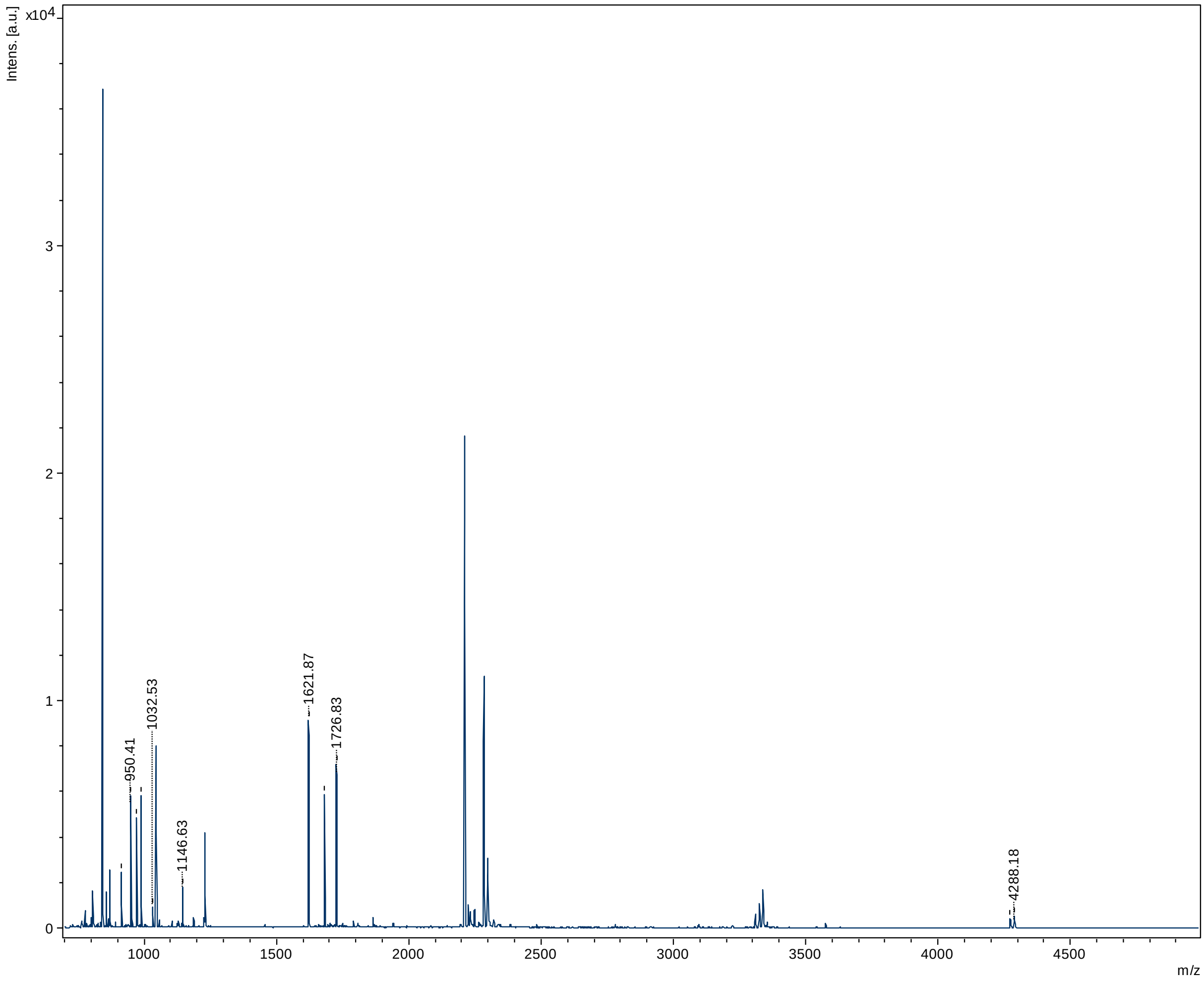

Identification:

gi|6320849 **Mass:** 22503 **Score:** 145 **Expect:** 5.7e-08 **Matches:** 9

Pre1p [Saccharomyces cerevisiae S288c]

Observed	Mr(expt)	Mr(calc)	ppm	Start		End	Miss	Peptide
915.5216	914.5143	914.5185	-4.62	183	-	190	1	K.IVDKDGIR.Q
950.4120	949.4047	949.4141	-9.92	191	-	198	0	R.QVDDFQAQ.-
1032.5271	1031.5198	1031.5321	-11.91	163	-	170	0	K.LCVQELEK.R + Propionamide (C)
1146.6278	1145.6205	1145.6292	-7.61	9	-	19	0	R.VQDSVILASSK.A
1621.8742	1620.8670	1620.8624	2.83	96	-	109	0	R.RPYQVNVLIGGYDK.K
1681.8746	1680.8674	1680.8723	-2.92	112	-	125	0	K.NKPELYQIDYLGTK.V
1726.8333	1725.8261	1725.8210	2.95	71	-	85	0	R.EDYELSPQAVSSFVR.Q
4272.1755	4271.1682	4271.0310	32.1	126	-	162	0	K.VELPYGAHGYSGFYTFSLLDHHYRPDMTTEEGLDLLK.L
4288.1835	4287.1762	4287.0259	35.1	126	-	162	0	K.VELPYGAHGYSGFYTFSLLDHHYRPDMTTEEGLDLLK.L + Oxidation (M)

No match to: 972.5487, 988.5405

Pre2

Mass-spectrum:

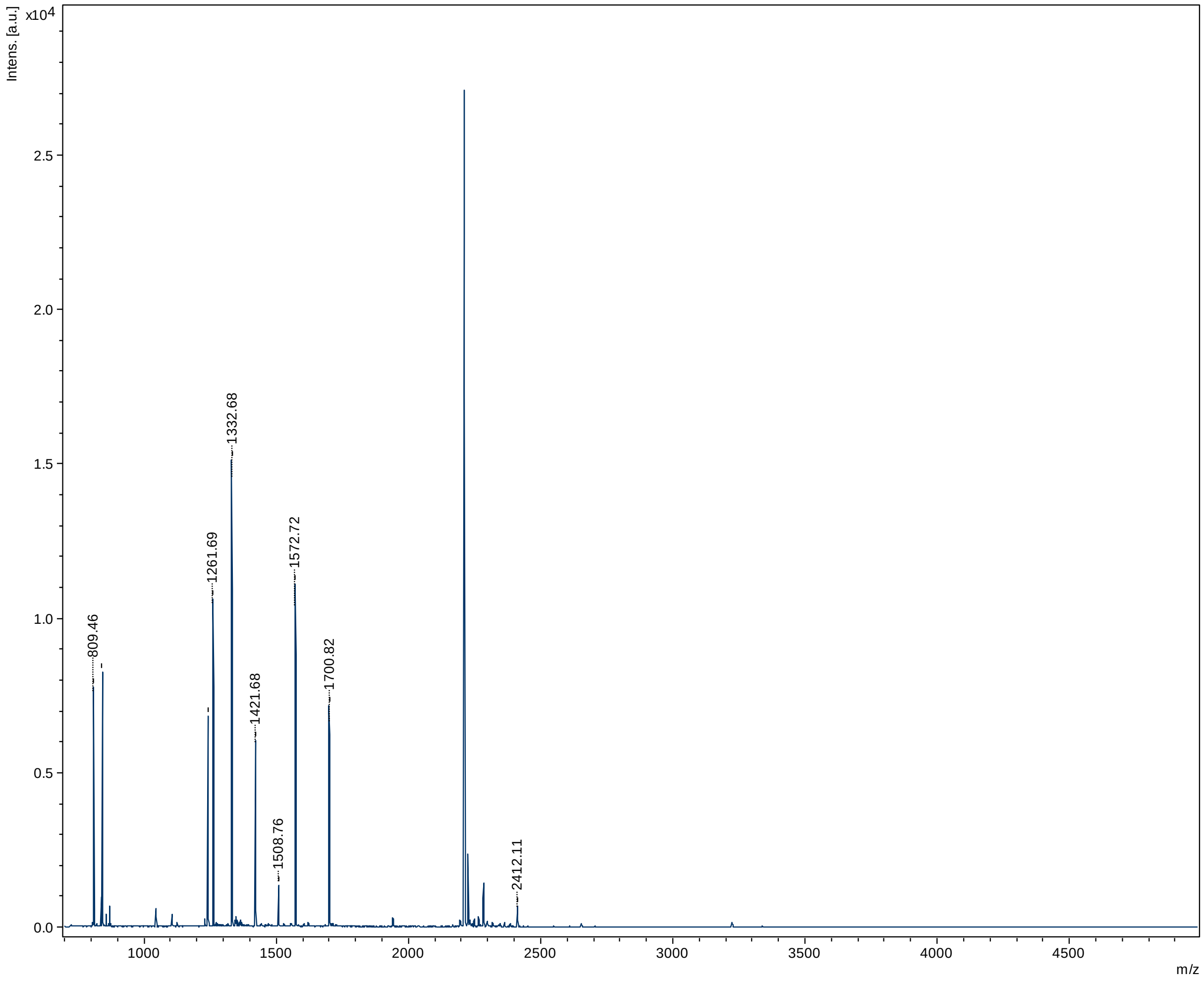

Identification:

gi|11514002 **Mass:** 23286 **Score:** **194** **Expect:** 7.2e-13 **Matches:** 10

Chain K, A Gated Channel Into The Proteasome Core Particle

Observed	Mr(expt)	Mr(calc)	ppm	Start		End	Miss	Peptide
809.4560	808.4487	808.4443	5.46	1	-	7	0	-.TTTLAFR.F
838.4897	837.4824	837.4821	0.40	160	-	167	0	R.SILAAAHR.D
1240.6845	1239.6772	1239.6863	-7.34	82	-	91	0	K.ILSNLVYQYK.G
1261.6886	1260.6813	1260.6826	-1.05	8	-	19	0	R.FQGGIIVAVDSR.A
1332.6844	1331.6771	1331.6834	-4.70	20	-	32	0	R.ATAGNWVASQTVK.K
1421.6790	1420.6717	1420.6834	-8.22	200	-	212	1	K.VKEEEGSFNNVIG.-
1508.7573	1507.7500	1507.7558	-3.86	146	-	158	0	K.WDLSVEDALYLGK.R
1572.7225	1571.7152	1571.7104	3.07	108	-	121	0	K.EGPTIYYVDSDGTR.L
1700.8175	1699.8102	1699.8053	2.87	107	-	121	1	R.KEGPTIYYVDSDGTR.L
2412.1086	2411.1013	2411.1104	-3.77	124	-	145	0	K.GDIFCVGSGQTFAYGVLDSNYK.W + Propionamide

Pre3

Mass-spectrum:

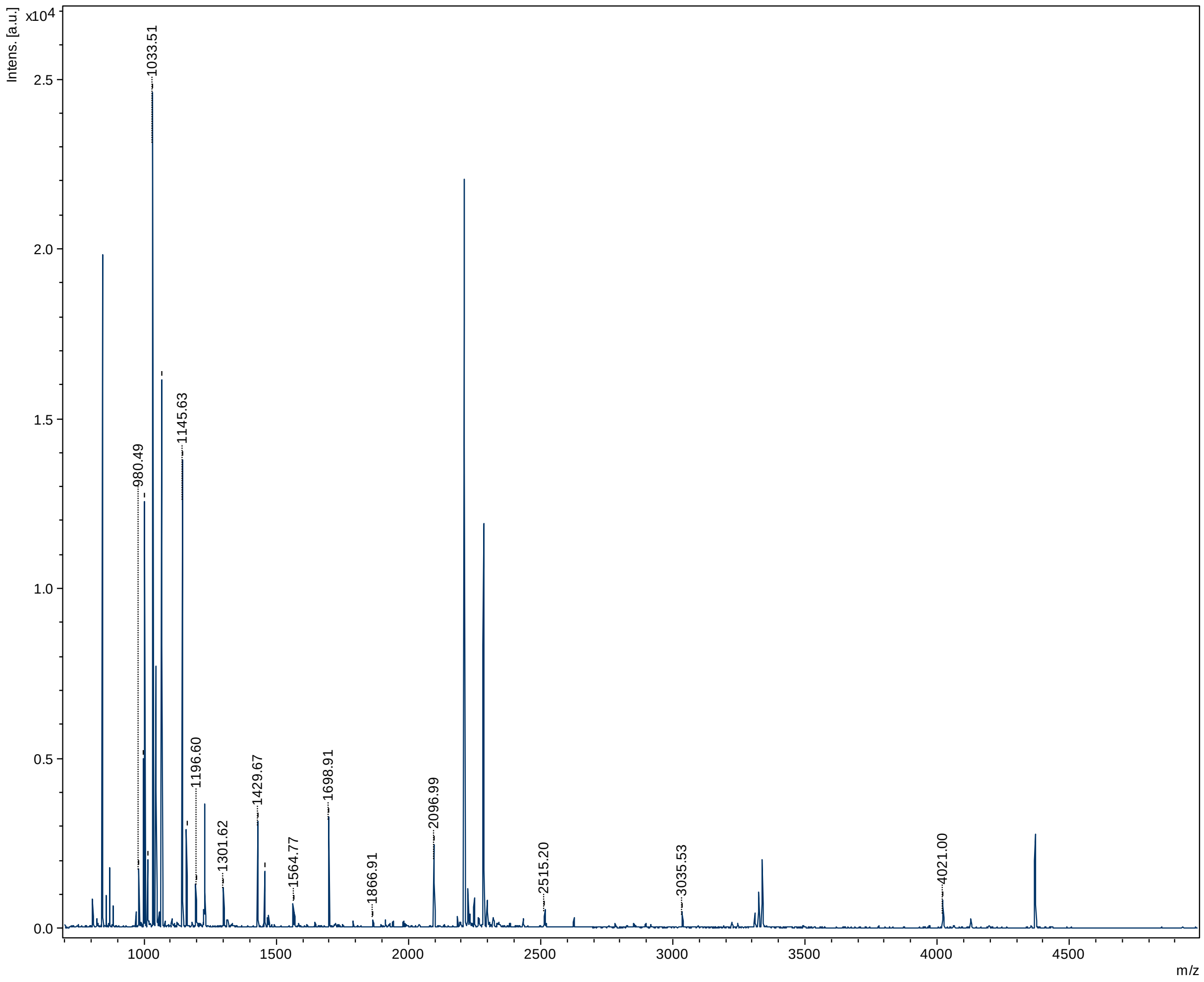

Identification:

gi|11513426 **Mass:** 21481 **Score:** **251** **Expect:** 1.4e-18 **Matches:** 16

Chain N, Crystal Structure Of Epoxomicin:20s Proteasome Reveals A Molecular Basis For Selectivity Of Alpha,Beta-Epoxyke

**Observed**	**Mr(expt)**	**Mr(calc)**	**ppm**	**Start**	**End**	**Miss**	**Peptide**
980.4863	979.4790	979.4862	-7.34	149 -	156	0	K.EETVDFIK.H
997.5306	996.5233	996.5314	-8.14	1 -	9	0	-.TSIMAVTFK.D
1002.5243	1001.5171	1001.5142	2.89	10 -	19	0	K.DGVILGADSR.T
1013.5226	1012.5153	1012.5263	-10.86	1 -	9	0	-.TSIMAVTFK.D + Oxidation (M)
1033.5107	1032.5035	1032.4989	4.44	165 -	174	0	K.WDGSSGGVIR.M
1067.5549	1066.5477	1066.5407	6.52	20 -	29	0	R.TTTGAYIANR.V
1145.6336	1144.6263	1144.6274	-0.97	175 -	185	0	R.MVVLTAAGVER.L
1161.6216	1160.6143	1160.6223	-6.93	175 -	185	0	R.MVVLTAAGVER.L + Oxidation (M)
1301.6247	1300.6174	1300.6169	0.39	37 -	45	1	R.VHDKIWCCR.S + 2 Propionamide (C)
1429.6718	1428.6645	1428.6813	-11.75	186 -	196	0	R.LIFYPDEYEQL.-
1456.7750	1455.7677	1455.7722	-3.09	108 -	121	0	K.GEVYTIPLGGSVHK.L
1564.7690	1563.7617	1563.7780	-10.45	91 -	105	0	K.DNLTAGIIVAGYDDK.N
1698.9108	1697.9035	1697.9101	-3.86	106 -	121	1	K.NKGEVYTIPLGGSVHK.L
2096.9863	2095.9790	2095.9925	-6.42	122 -	140	0	K.LPYAIAGSGSTFIYGYCDK.N + Propionamide (C)
2515.1950	2514.1878	2514.1948	-2.79	84 -	105	1	K.ELCYENKDNLTAGIIVAGYDDK.N + Propionamide (C)
4020.9992	4019.9919	4019.9276	16.0	46 -	83	0	R.SGSAADTQAIADIVQYHLELYTSQYGTPSTETAASVFK.E

No match to: 1196.5962, 1866.9107, 3035.5264

Pre4

Mass-spectrum:

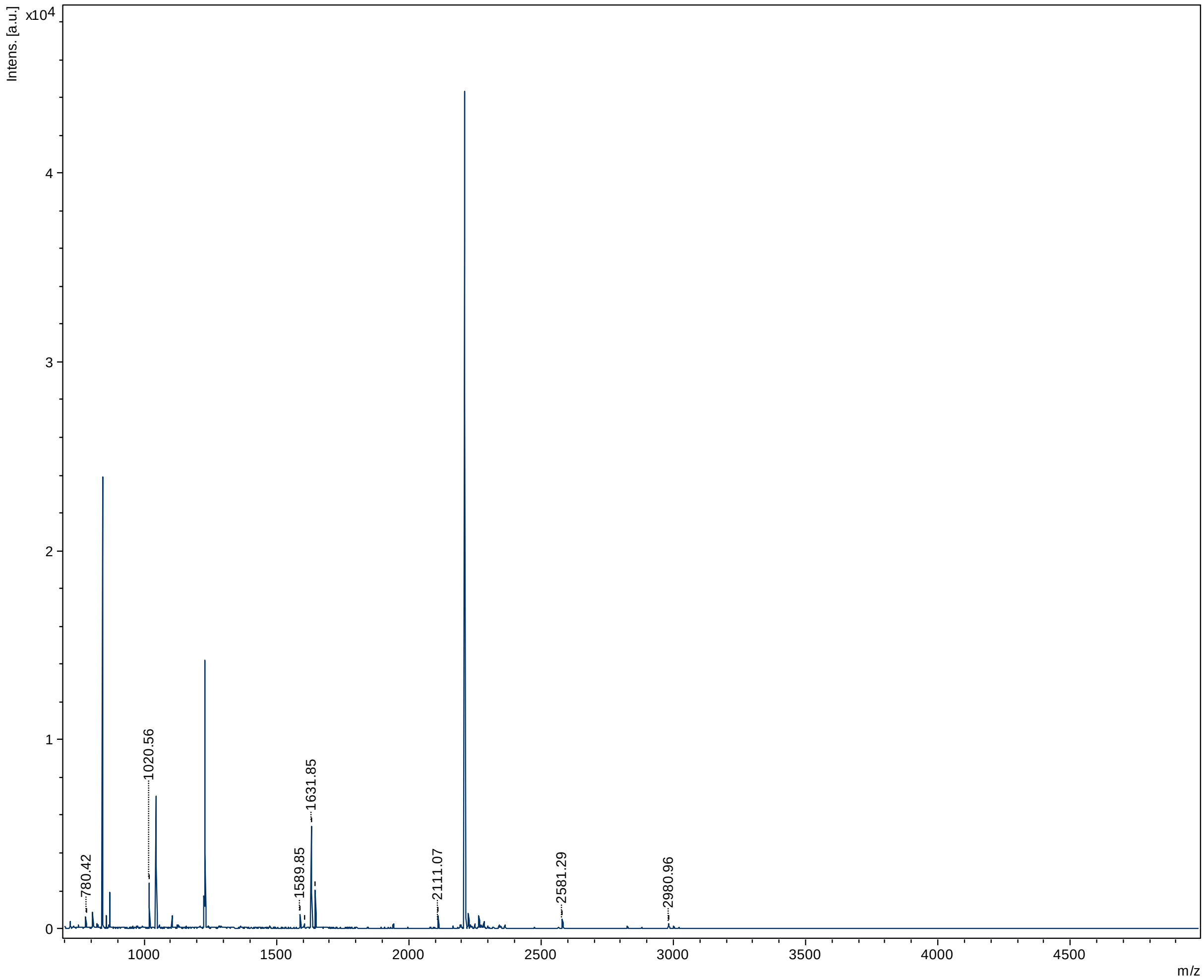

Identification:

gi|3114282 **Mass:** 25903 **Score:** **109** **Expect:** 0.00023 **Matches:** 8

Chain N, Crystal Structure Of The 20s Proteasome From Yeast At 2.4 Angstroms Resolution

Observed	Mr(expt)	Mr(calc)	ppm	Start		End	Miss	Peptide
780.4249	779.4177	779.4178	-0.13	203	-	209	0	K.NTGLTFK.K
1020.5641	1019.5568	1019.5651	-8.16	194	-	202	0	R.NFSLAIIDK.N
1589.8452	1588.8379	1588.8495	-7.29	1	-	15	0	-.TQQPIVTGTSVISMK.Y
1605.8338	1604.8265	1604.8444	-11.13	1	-	15	0	-.TQQPIVTGTSVISMK.Y + Oxidation (M)
1631.8465	1630.8393	1630.8348	2.71	168	-	182	0	K.TTVQVAEEAIVNAMR.V
1647.8321	1646.8249	1646.8298	-2.97	168	-	182	0	K.TTVQVAEEAIVNAMR.V + Oxidation (M)
2111.0715	2110.0642	2110.0695	-2.47	16	-	35	0	K.YDNGVIIAADNLGSYGSLLR.F
2581.2919	2580.2847	2580.2854	-0.28	42	-	65	0	R.LIPVGDNTVVGISGDISDMQHIER.L + Oxidation (M)

No match to: 2980.9648

Pre6

Mass-spectrum:

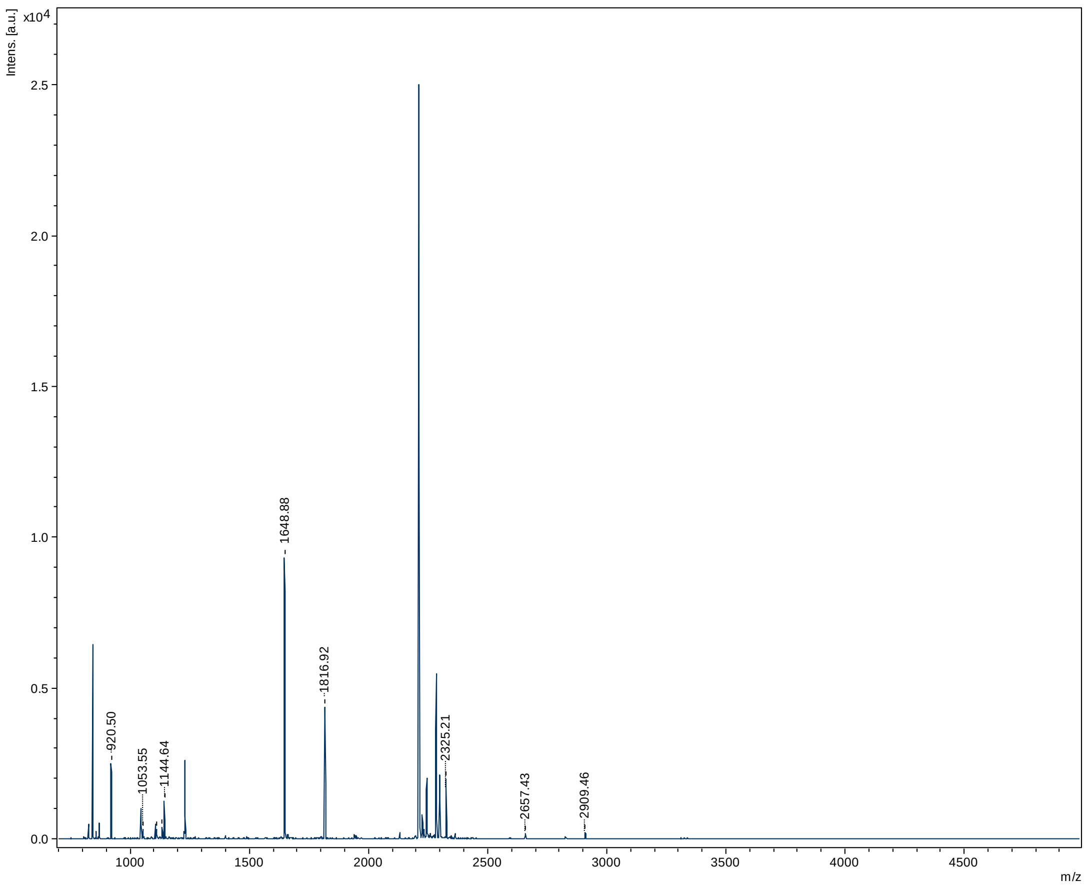

Identification:

gi|298508225 **Mass:** 25337 **Score:** 151 **Expect:** 1.4e-08 **Matches:** 9

Chain D, Proteasome Activator Complex

Observed	Mr(expt)	Mr(calc)	ppm	Start	End	Miss	Peptide
920.4969	919.4897	919.4876	2.27	96 -	103	0	R.YVAGVQQR.Y
1053.5469	1052.5396	1052.5475	-7.49	73 -	81	1	K.ARVEAQSHR.L
1134.5359	1133.5286	1133.5321	-3.12	24 -	32	0	K.NCVVLGCER.R + 2 Propionamide (C)
1144.6443	1143.6370	1143.6499	-11.29	182 -	192	0	R.SLLEVVQTGAK.N
1648.8790	1647.8717	1647.8719	-0.14	82 -	95	0	R.LTLEDPVTVEYLTR.Y
1816.9241	1815.9168	1815.9115	2.91	51 -	67	0	K.IDSHVVLSFSGLNADSR.I
2325.2071	2324.1998	2324.1914	3.63	104 -	125	0	R.YTQSGGVRPFGVSTLIAGFDPR.D
2657.4348	2656.4276	2656.4184	3.44	43 -	67	2	R.ITPSKVSKIDSHVVLSFSGLNADSR.I
2909.4633	2908.4560	2908.4356	7.04	104 -	130	1	R.YTQSGGVRPFGVSTLIAGFDPRDDEPK.L

No match to: 1111.5450

Pre7

Mass-spectrum:

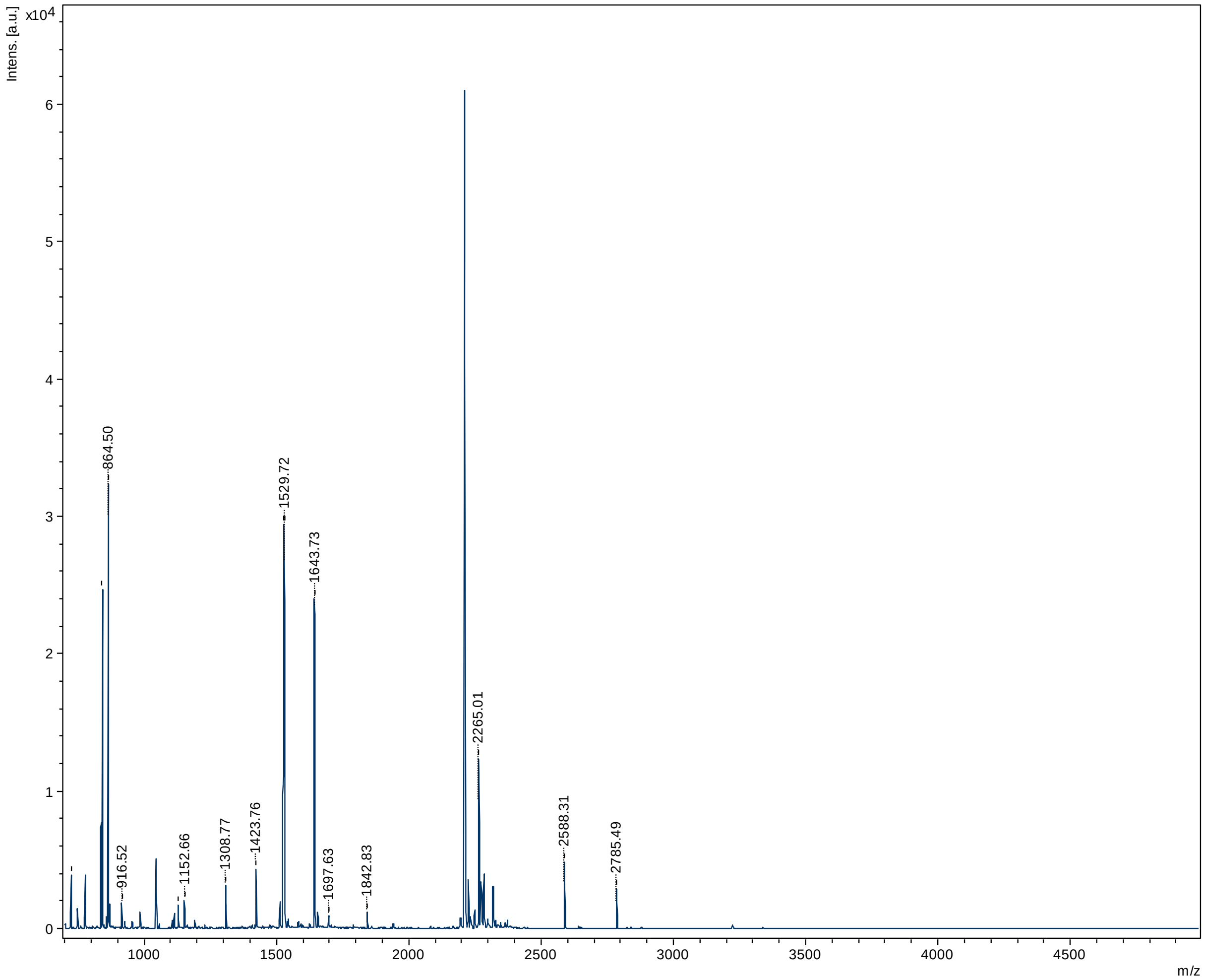

Identification:

gi|3114281 **Mass:** 24836 **Score:** **120** **Expect:** 1.8e-05 **Matches:** 7

Chain M, Crystal Structure Of The 20s Proteasome From Yeast At 2.4 Angstroms Resolution

Observed	Mr(expt)	Mr(calc)	ppm	Start	End	Miss	Peptide
831.4664	830.4591	830.4610	-2.21	84 -	91	0	K.LSINSAAR.N
1013.4514	1012.4442	1012.4462	-1.96	186 -	194	0	R.DSFTSATER.H
1079.5887	1078.5814	1078.5910	-8.86	174 -	182	0	K.YLSVEEVIK.L
1182.5769	1181.5696	1181.5676	1.65	29 -	38	0	R.NITDYSINSR.Y
1546.7313	1545.7240	1545.7100	9.09	120 -	133	0	K.GAVYSFDPVGSYER.E
2080.0432	2079.0359	2079.0459	-4.80	138 -	157	0	R.AGGAAASLIMPFLDNQVNFK.N + Oxidation (M)
2516.1182	2515.1109	2515.1359	-9.94	43 -	66	0	K.VFDCGDNIVMSANGFAADGDALVK.R + Oxidation (M); Propionamide (C)

No match to: 2265.0097, 2851.3574

Pre8

Mass-spectrum:

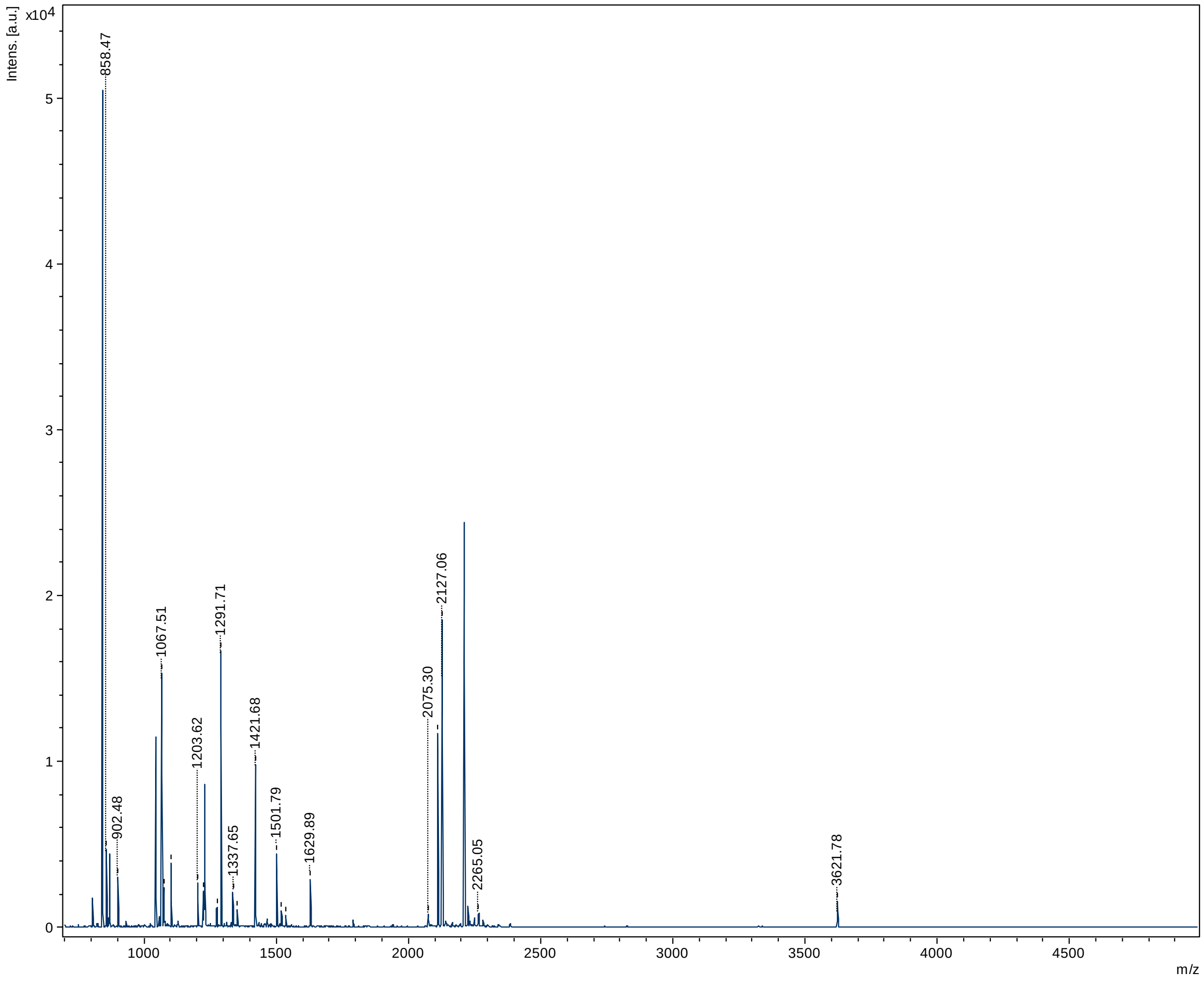

Identification:

gi|6323547 **Mass:** 27145 **Score:** 550 **Expect:** 1.8e-48 **Matches:** 15

Pre8p [Saccharomyces cerevisiae S288c]

Observed	Mr(expt)	Mr(calc)	ppm	Start	End	Miss	Ions	Peptide
858.4728	857.4656	857.5222	-66.04	109 -	116	0	---	K.LLVSEVAK.I
902.4785	901.4712	901.5233	-57.73	30 -	38	0	---	K.QGVTSLGIK.A
1067.5088	1066.5015	1066.5335	-29.97	100 -	108	0	54	R.IYGEYPPTK.L
1075.5131	1074.5058	1074.5305	-23.02	238 -	246	0	---	K.LTSQEINDR.L
1102.5759	1101.5686	1101.6030	-31.18	39 -	49	0	---	K.ATNGVVIATEK.K
1203.6157	1202.6084	1202.6255	-14.20	237 -	246	1	---	R.KLTSQEINDR.L
1223.6262	1222.6189	1222.6346	-12.82	99 -	108	1	---	K.RIYGEYPPTK.L
1291.7081	1290.7008	1290.7183	-13.60	18 -	29	0	78	K.LGQIDYALTAVK.Q
1337.6480	1336.6407	1336.6544	-10.25	51 -	63	0	---	K.SSSPLAMSETLSK.V
1353.6402	1352.6330	1352.6493	-12.09	51 -	63	0	---	K.SSSPLAMSETLSK.V + Oxidation (M)
1421.6838	1420.6766	1420.6875	-7.67	5 -	17	0	79	R.YSFSLTTFSPSGK.L
1501.7880	1500.7808	1500.7784	1.60	238 -	250	1	---	K.LTSQEINDRLEAL.-
2111.0600	2110.0527	2110.0405	5.78	64 -	83	0	139	K.VSLLTPDIGAVYSGMGPDYR.V
2127.0580	2126.0507	2126.0354	7.19	64 -	83	0	---	K.VSLLTPDIGAVYSGMGPDYR.V + Oxidation (M)
3621.7793	3620.7720	3620.7257	12.8	198 -	231	0	---	K.ESVEGEFNGDTIELAIIGDENPDLLGYTGIPTDK.G

No match to: 1276.6245, 1520.7460, 1536.7419, 1629.8904, 2075.2977, 2265.0508

Pre9

Mass-spectrum:

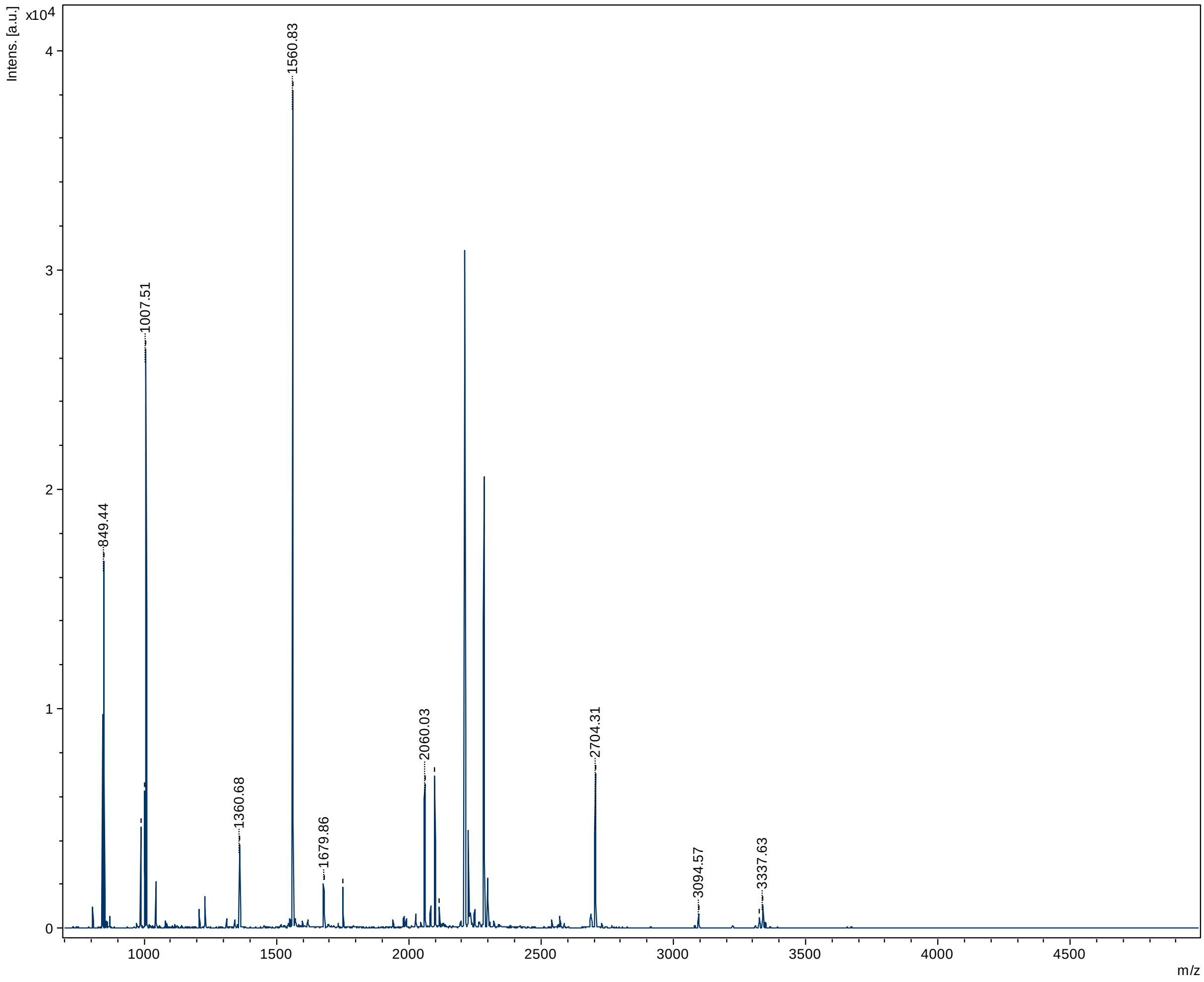

Identification:

gi|3114271   **Mass:** 27003   **Score:** **467**   **Expect:** 3.6e-40   **Matches:** 9

Chain C, Crystal Structure Of The 20s Proteasome From Yeast At 2.4 Angstroms Resolution

Observed	Mr(expt)	Mr(calc)	ppm	Start	End	Miss	Ions	Peptide
849.4430	848.4357	848.4756	-46.95	210 -	216	0	---	R.LEFATIR.K
986.5208	985.5135	985.5345	-21.30	92 -	99	0	---	R.IHAQNYLK.T
1002.5741	1001.5668	1001.5909	-24.09	228 -	235	0	---	K.IFKPQEIK.D
1007.5056	1006.4983	1006.5084	-9.98	9 -	17	0	46	R.TTIFSPEGR.L
1560.8267	1559.8195	1559.8195	-0.03	100 -	112	0	99	K.TYNEDIPVEILVR.R
1679.8610	1678.8537	1678.8625	-5.25	50 -	64	1	---	R.KVTSTLLEQDTSTEK.L
2060.0345	2059.0273	2059.0222	2.47	199 -	216	1	60	K.TTDSSALTYDRLEFATIR.K
2098.9581	2097.9508	2097.9432	3.62	143 -	160	0	132	R.YGYQLYTSNPSGNYTGWK.A
2704.3071	2703.2998	2703.2830	6.20	119 -	142	0	---	K.QGYTQHGGLRPFGVSFIYAGYDDR.Y

No match to: 1360.6763, 1362.5970, 1750.8711, 2114.9560, 3094.5718, 3323.6187, 3337.6344

Pup2

Mass-spectrum:

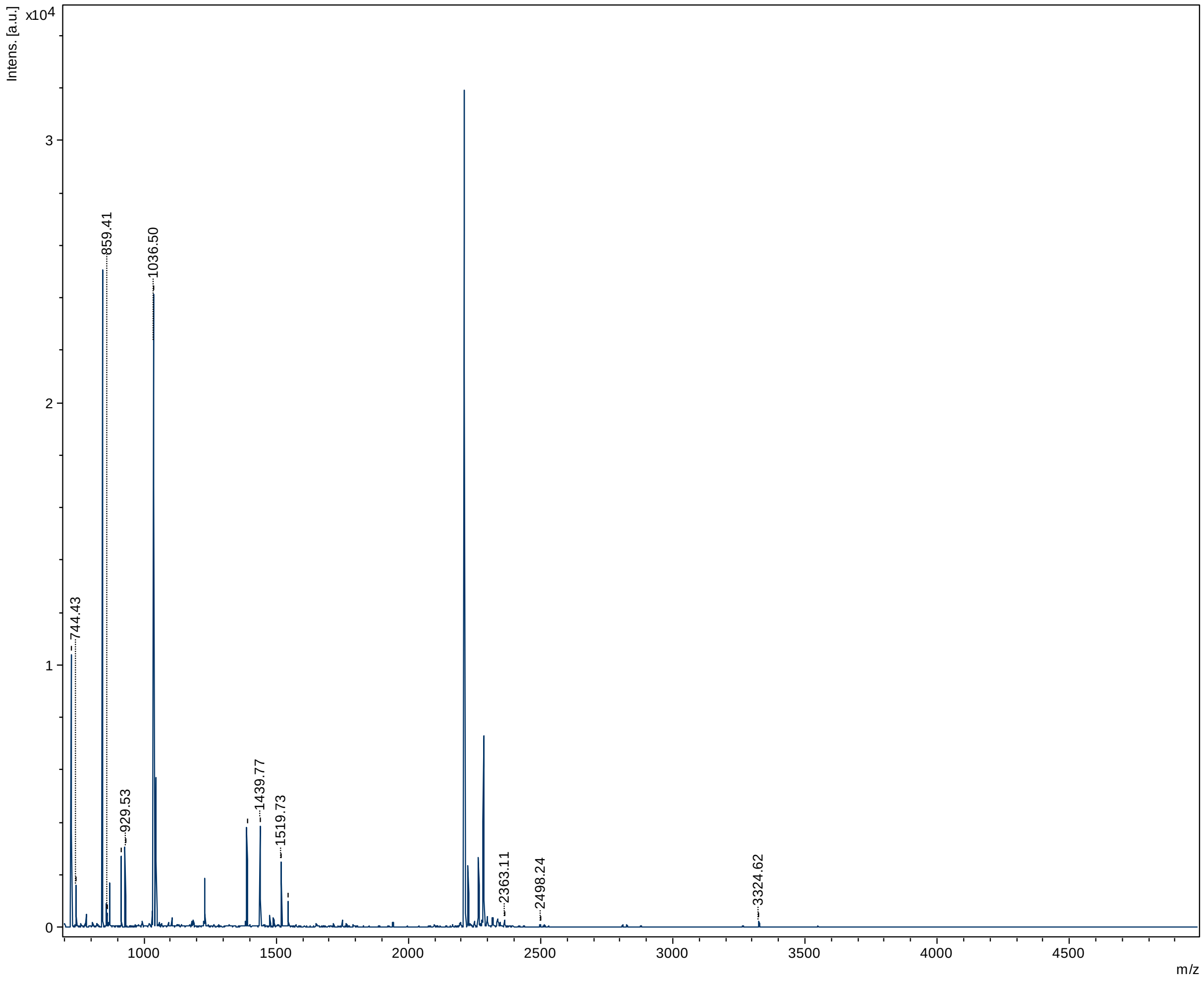

Identification:

gi|298508226 **Mass:** 27525 **Score:** 217 **Expect:** 3.6e-15 **Matches:** 13

Chain E, Proteasome Activator Complex

Observed	Mr(expt)	Mr(calc)	ppm	Start	End	Miss	Peptide
725.3733	724.3660	724.3578	11.4	1	5	0	-.MFLTR.S + Acetyl (Protein N-term); Oxidation (M)
744.4316	743.4243	743.4177	8.88	67	72	0	K.IVEIDR.H
859.4103	858.4030	858.4018	1.45	87	93	0	R.SMIEHAR.T + Oxidation (M)
914.5503	913.5430	913.5484	-5.91	195	202	0	K.EAELLVLK.I
929.5254	928.5181	928.5229	-5.23	44	52	0	K.EGVVLGVEK.R
1036.4968	1035.4895	1035.4985	-8.71	11	20	0	R.GVSTFSPEGR.L
1038.4456	1037.4383	1037.4414	-3.00	123	132	0	R.FGEGASGEER.L
1389.7084	1388.7012	1388.7035	-1.66	54	66	0	R.ATSPLLESDSIEK.I
1439.7723	1438.7651	1438.7708	-3.95	21	32	0	R.LFQVEYSLEAIK.L
1519.7349	1518.7276	1518.7348	-4.73	212	224	0	K.LDENNAQLSCITK.Q + Propionamide (C)
1545.8164	1544.8092	1544.8046	2.98	53	66	1	K.RATSPLLESDSIEK.I
2498.2448	2497.2375	2497.2448	-2.93	171	194	0	K.AIGSGSEGAQAELLNEWHSSLTLK.E
3324.6246	3323.6173	3323.5980	5.81	94	122	0	R.TAAVTHNLYYDEDINVESLTQSVCDLALR.F + Propionamide (C)

No match to: 2363.1102

Pup3

Mass-spectrum:

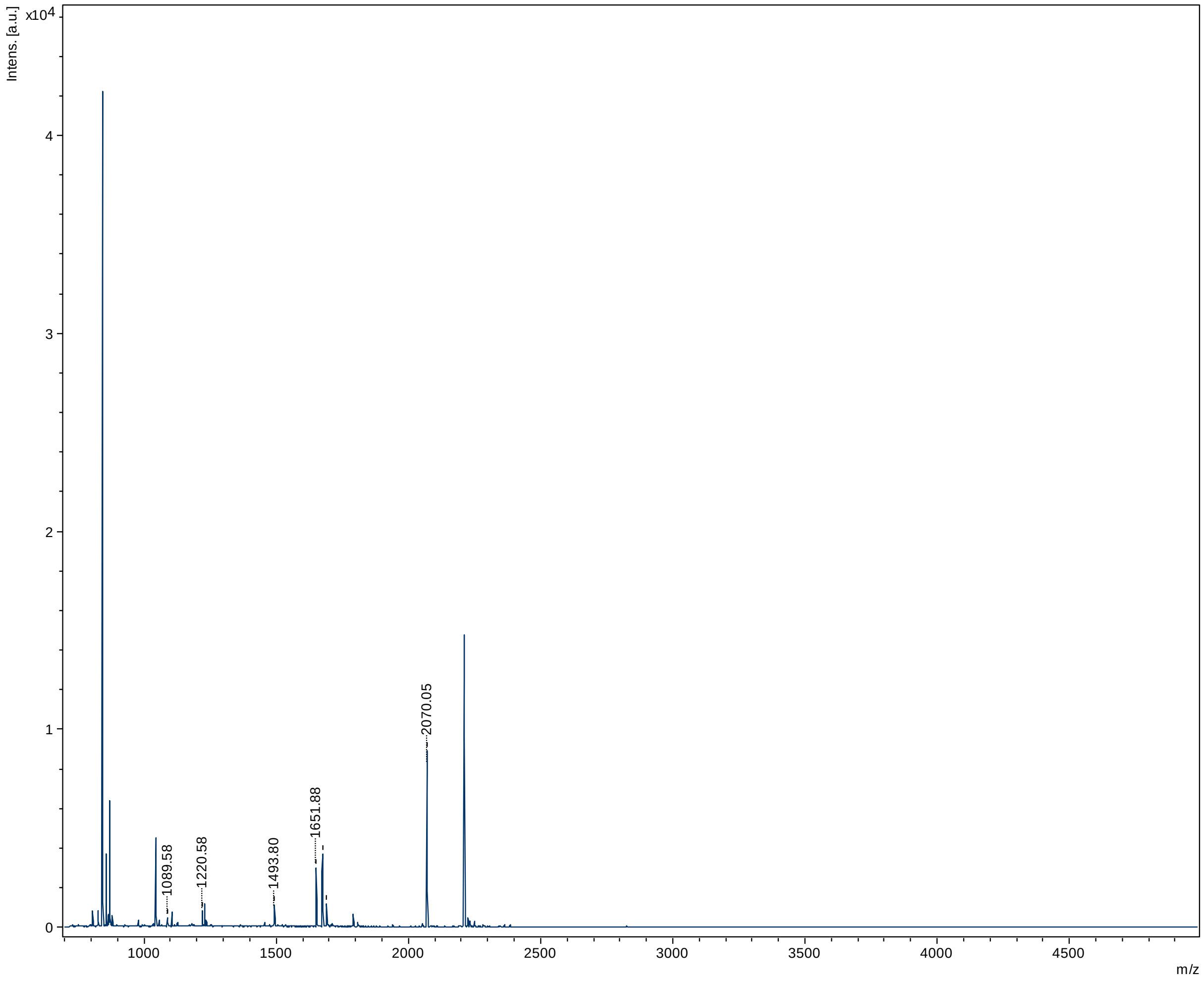

Identification:

gi|323309416 **Mass:** 21263 **Score:** 87 **Expect:** 0.039 **Matches:** 5

Pup3p [Saccharomyces cerevisiae FostersO]

Observed	Mr(expt)	Mr(calc)	ppm	Start		End	Miss	Peptide
1089.5814	1088.5742	1088.5826	-7.73	15	-	25	0	R.LGSQSLGVSNK.F
1220.5782	1219.5709	1219.5689	1.64	5	-	14	0	K.DCVAIACDLR.L + 2 Propionamide (C)
1493.7990	1492.7918	1492.7885	2.16	15	-	28	1	R.LGSQSLGVSNKFEK.I
1651.8803	1650.8730	1650.8770	-2.42	86	-	101	0	R.FGPYFVGPVVAGINSK.S
2070.0509	2069.0436	2069.0317	5.78	67	-	84	0	R.AIEPETFTQLVSSSLYER.R

No match to: 1674.8348, 1690.8224

Put2

Mass-spectrum:

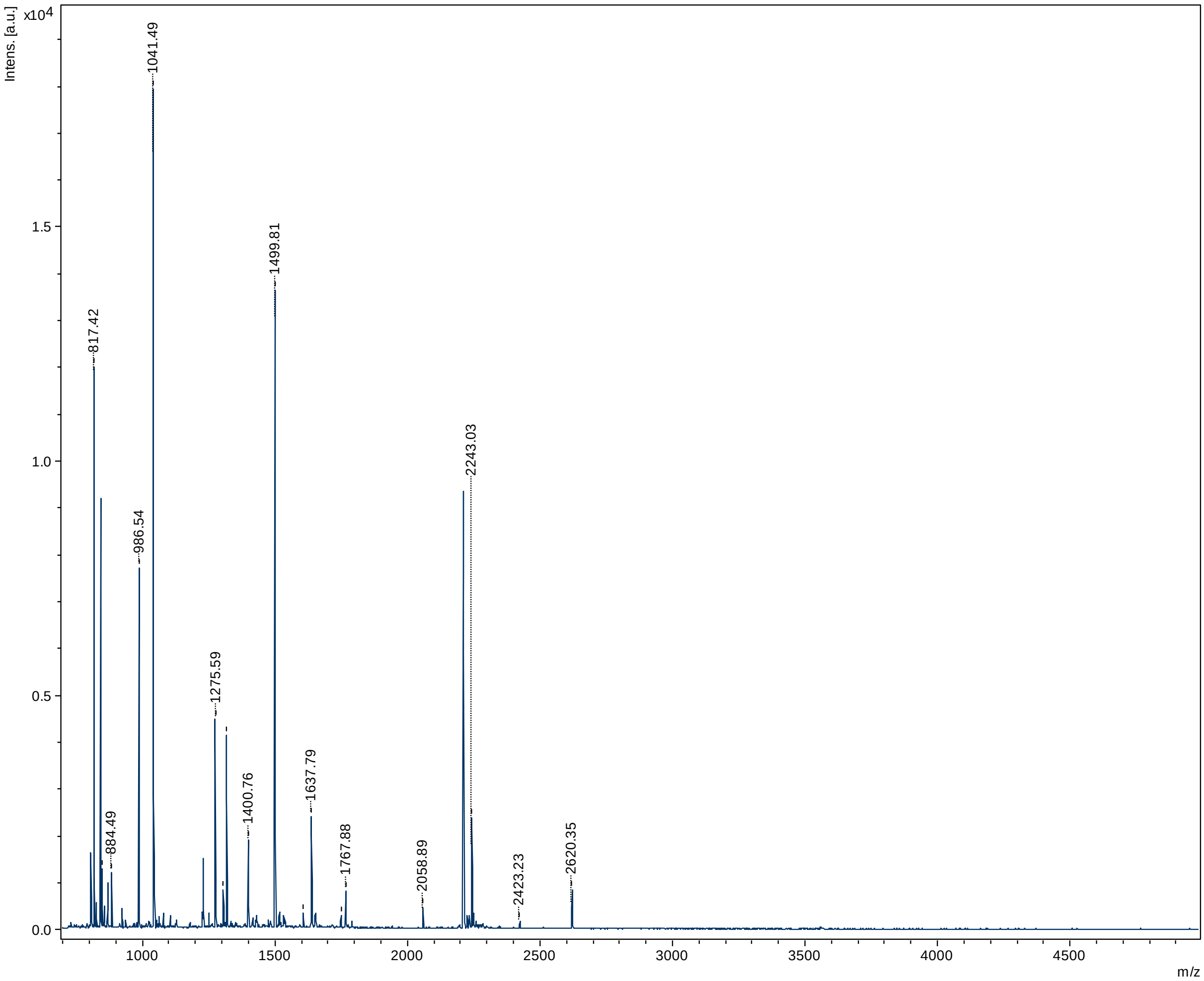

Identification:

gi|6321826 **Mass:** 64394 **Score:** **247** **Expect:** 3.6e-18 **Matches:** 17

Put2p [Saccharomyces cerevisiae S288c]

Observed	Mr(expt)	Mr(calc)	ppm	Start	End	Miss	Peptide
817.4177	816.4104	816.4130	-3.20	43 -	48	0	K.DWDLLR.A
849.4580	848.4508	848.4643	-16.00	357 -	363	0	R.LYLPESK.S
884.4942	883.4869	883.4875	-0.71	543 -	551	0	K.AGGPNILSR.F
986.5383	985.5311	985.5345	-3.50	30 -	37	0	R.NEPVKPFR.N
1041.4886	1040.4813	1040.4927	-10.97	342 -	350	0	R.GTFEFQGQK.C
1275.5941	1274.5869	1274.5931	-4.93	510 -	520	0	K.FSAGNFYINDK.C
1305.5934	1304.5862	1304.5925	-4.83	560 -	569	0	K.ENFYELTDFK.Y
1318.7103	1317.7030	1317.7081	-3.89	435 -	446	0	K.SQGWFVGPTVIK.A
1400.7576	1399.7504	1399.7459	3.16	38 -	48	1	R.NIDLKDWDLLR.A
1499.8105	1498.8032	1498.7991	2.76	56 -	69	0	K.SSSLEVPLVINGER.I
1607.7435	1606.7363	1606.7450	-5.44	397 -	410	0	R.GFMGPVIHEQSFDK.L + Oxidation (M)
1637.7946	1636.7873	1636.7780	5.65	521 -	535	0	K.CTGAVVSQQWFGGAR.M + Propionamide (C)
1749.8315	1748.8242	1748.8226	0.94	139 -	153	1	K.YRYDMLAATMLGQGK.N + 2 Oxidation (M)
1767.8823	1766.8750	1766.8727	1.32	420 -	434	1	K.KDPELEILYGGQYDK.S
2058.8901	2057.8828	2057.8894	-3.22	560 -	575	1	K.ENFYELTDFKYPSNYE.-
2243.0261	2242.0189	2242.0178	0.46	176 -	195	0	K.YASDLYAQQPVESADGTWNK.A
2620.3509	2619.3436	2619.3405	1.19	77 -	100	0	R.ALFPQTNPANHQQVLANVTQATEK.D

No match to: 2423.2348

Scl1

Mass-spectrum:

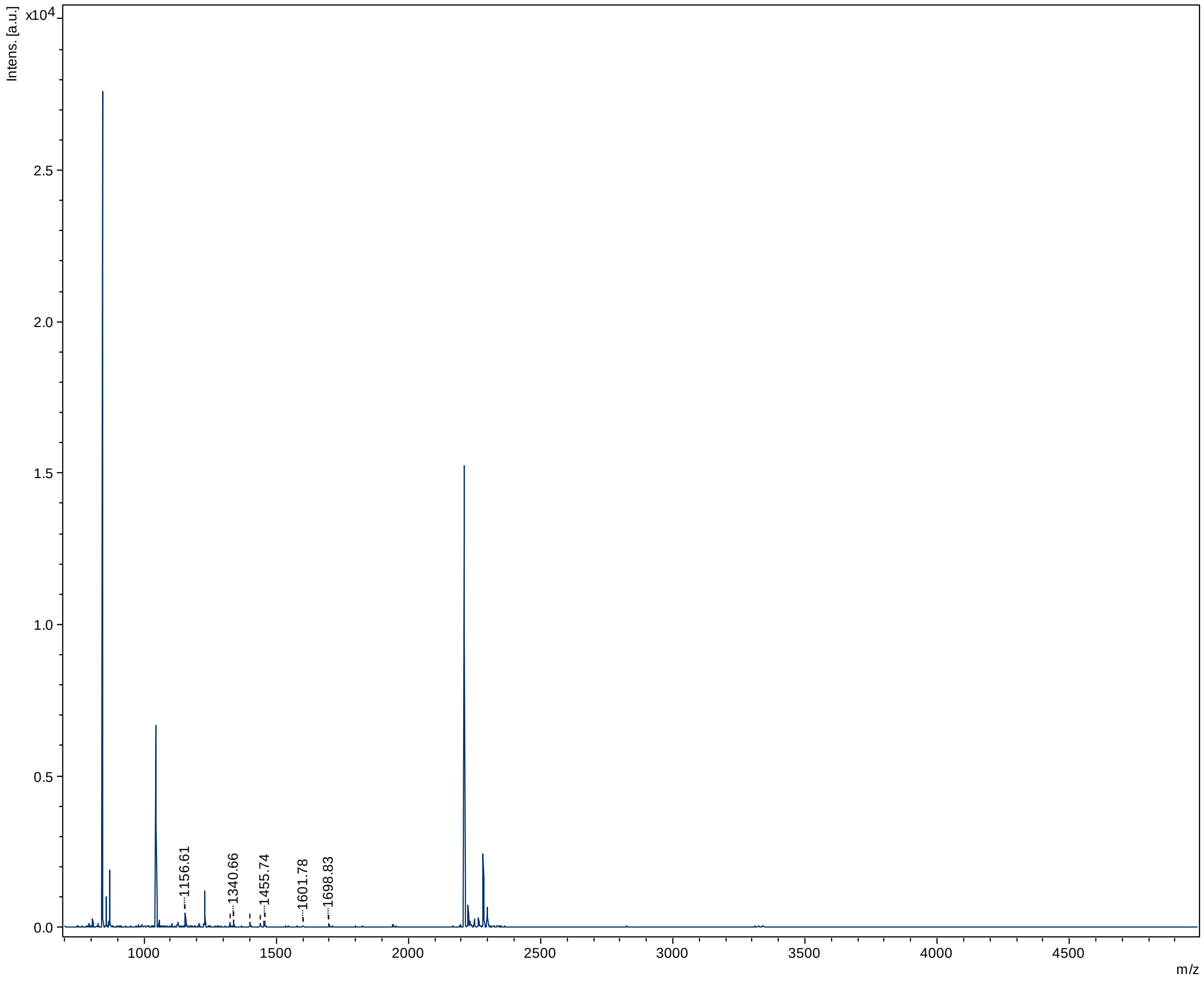

Identification:

gi|6321427 27983110 **Score: 110** **Expect:** 0.00018 **Matches:** 8

Scl1p [Saccharomyces cerevisiae S288c]

Observed	Mr(expt)	Mr(calc)	ppm	Start	End	Miss	Peptide
1156.6060	1155.5987	1155.6037	-4.30	15	24	0	R.HITIFSPEGR.L
1324.6688	1323.6616	1323.6605	0.81	121	131	0	R.MANLSQIYTQR.A
1340.6577	1339.6505	1339.6554	-3.69	121	131	0	R.MANLSQIYTQR.A + Oxidation (M)
1401.7342	1400.7269	1400.7372	-7.34	34	46	0	K.ATNQTNINSLAVR.G
1439.7653	1438.7581	1438.7602	-1.52	78	91	0	R.TIGMVVNGPIPDAR.N
1455.7399	1454.7326	1454.7552	-15.50	78	91	0	R.TIGMVVNGPIPDAR.N + Oxidation (M)
1601.7799	1600.7726	1600.7845	-7.45	175	187	0	K.QQEITTNLENHFK.K
1698.8347	1697.8274	1697.8260	0.83	231	244	1	K.DKFFTLSAENIEER.L
