# Supplementary material for: Proteomic Screening for Amyloid Proteins
Source: PLoS One. 2014 Dec 30;9(12):e116003. doi: 10.1371/journal.pone.0116003 (PMC4280166; doi:10.1371/journal.pone.0116003)
Supplement: S4 Table — Identification data of the proteins listed in Table 4. (PDF) [file pone.0116003.s004.pdf]

Table S4. Identification data of the proteins listed in Table 4.

Nop1

Mass-spectrum:

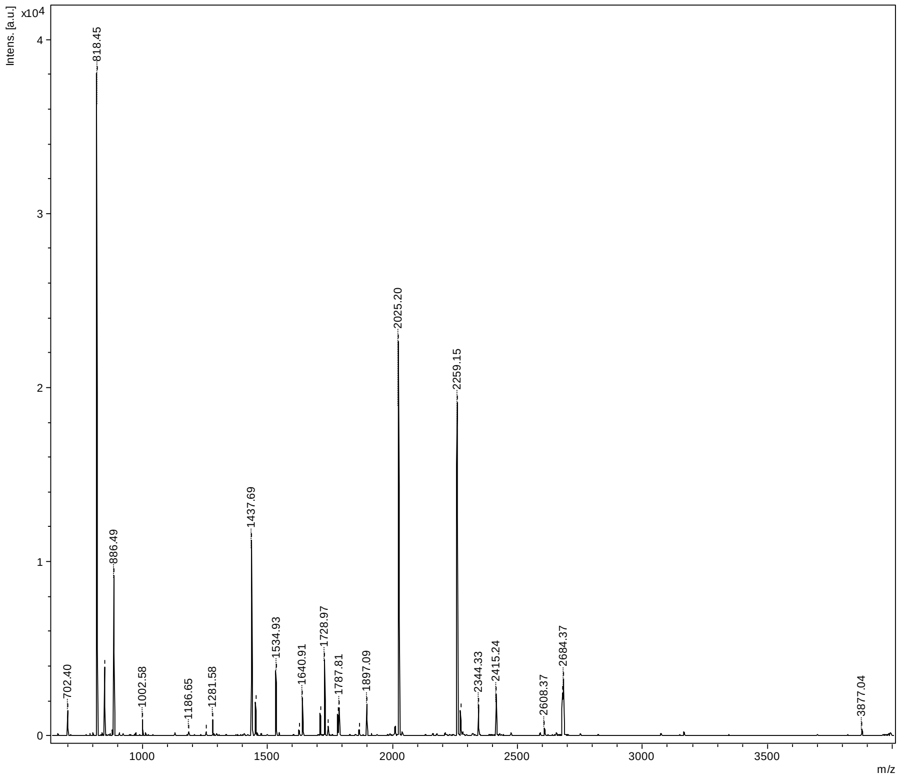

Identification:

gi|6320190 Mass: 34444 Score: 160 Expect: 4.5e-12 Matches: 15

Nop1p [Saccharomyces cerevisiae S288c]

Observed	Mr(expt)	Mr(calc)	ppm	Start		End	Miss	Peptide
702.4019	701.3946	701.3820	18.0	293	-	297	1	K.LREER.I
818.4471	817.4398	817.4235	19.9	143	-	148	0	R.VWNPFR.S
849.4919	848.4846	848.4868	-2.58	86	-	92	0	K.VVIEPHR.H
886.4877	885.4804	885.4821	-1.87	93	-	100	0	R.HAGVYIAR.G
1002.5778	1001.5705	1001.5757	-5.21	101	-	109	1	R.GKEDLLVTK.N
1281.5754	1280.5682	1280.5707	-1.97	110	-	121	0	K.NMAPGESVYGEK.R
1437.6856	1436.6783	1436.6718	4.57	110	-	122	1	K.NMAPGESVYGEKR.I
1453.6835	1452.6763	1452.6667	6.58	110	-	122	1	K.NMAPGESVYGEKR.I + Oxidation (M)
1534.9254	1533.9181	1533.8991	12.4	213	-	225	1	K.KRPNIIPIIEDAR.H
1711.8647	1710.8574	1710.8676	-5.93	123	-	138	1	R.ISVEEPSKEDGVPPTK.V
1728.9735	1727.9662	1727.9457	11.8	298	-	311	0	R.IKPLEQLTLEPYER.D
1782.8506	1781.8434	1781.8254	10.1	273	-	288	0	K.ANCIDSTVDAETVFAR.E + Propionamide (C)
1897.0908	1896.0835	1896.0693	7.50	214	-	229	1	K.RPNIIPIIEDARHPQK.Y
2608.3667	2607.3594	2607.3843	-9.52	298	-	319	1	R.IKPLEQLTLEPYERDHCIVVGR.Y
2679.4047	2678.3974	2678.4214	-8.95	298	-	319	1	R.IKPLEQLTLEPYERDHCIVVGR.Y + Propionamide (C)

No match to: 1186.6456, 1255.7002, 1626.8913, 1640.9100, 1742.9920, 1787.8113, 1867.9843, 2025.2024, 2259.1495, 2273.1772, 2344.3313, 2415.2420, 2684.3726, 3877.0421

Rpl28

Mass-spectrum:

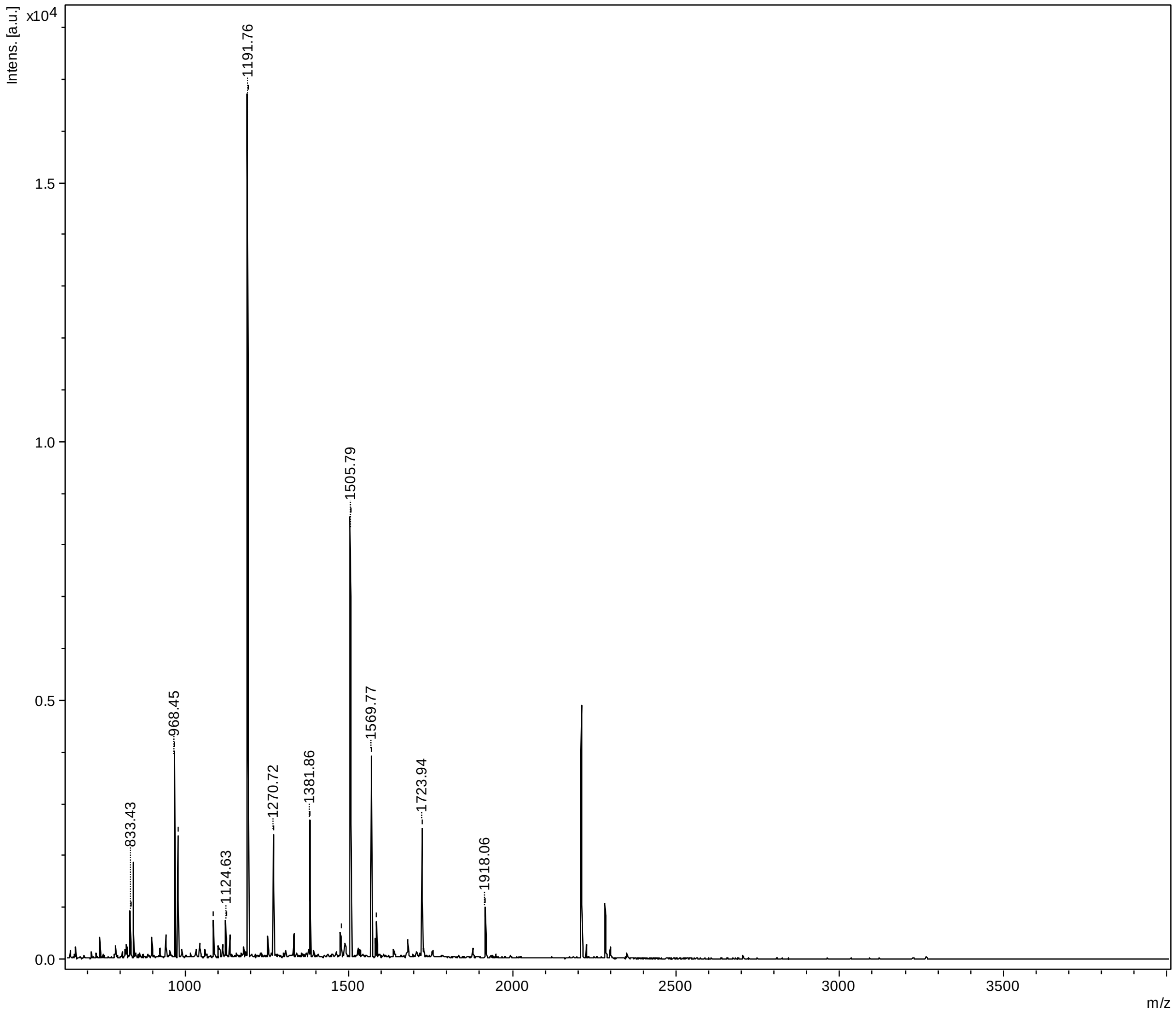

Identification:

gi|323305009 **Mass:** 13056 **Score:** 130 **Expect:** 3.3e-06 **Matches:** 8

Rpl28p [Saccharomyces cerevisiae FostersB]

Observed	Mr(expt)	Mr(calc)	ppm	Start		End	Miss	Peptide
968.4534	967.4462	967.4552	-9.35	15	-	22	0	K.YHPGYFGK.V
978.6203	977.6131	977.6273	-14.60	85	-	93	0	R.IPNVPVIVK.A
1191.7578	1190.7505	1190.7499	0.51	83	-	93	1	K.GRIPNVPVIVK.A
1270.7177	1269.7105	1269.7081	1.87	45	-	54	1	K.LWTLIPEDKR.D
1505.7904	1504.7831	1504.7773	3.84	64	-	78	0	K.ETAPVIDTLAAGYGK.I
1569.7660	1568.7587	1568.7446	9.01	10	-	22	1	R.INMDKYHPGYFGK.V
1585.7559	1584.7486	1584.7395	5.76	10	-	22	1	R.INMDKYHPGYFGK.V + Oxidation (M)
1723.9376	1722.9303	1722.9206	5.67	31	-	44	0	K.QQAHFWKPVLNLDK.L

No match to: 833.4252, 1087.5028, 1124.6289, 1381.8616, 1475.7669, 1918.0642

Rpl3

Mass-spectrum:

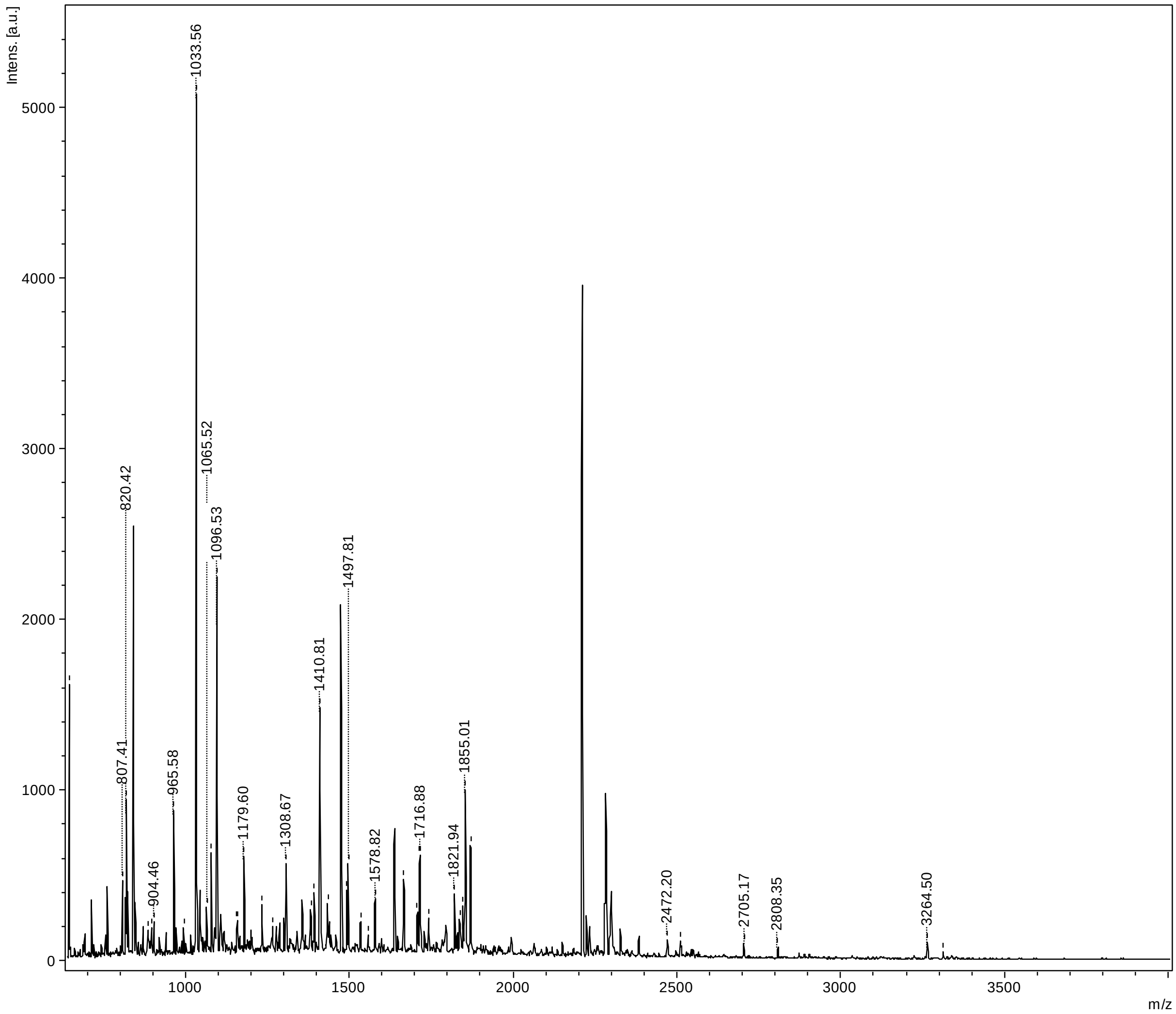

Identification:

gi|49258841 Mass: 43599 **Score: 88 Expect:** 0.048 **Matches:** 11

Chain C, Structure Of The Ribosomal 80s-Eef2-Sordarin Complex From Yeast Obtained By Docking Atomic Models For Rna

Observed	Mr(expt)	Mr(calc)	ppm	Start	End	Miss	Peptide
820.4208	819.4135	819.4127	1.04	369 -	375	0	R.FQTPAEK.H
904.4591	903.4518	903.4637	-13.08	376 -	383	0	K.HAFMGTLK.K
965.5845	964.5772	964.5818	-4.74	159 -	166	0	R.VLVHTQIR.K
1033.5613	1032.5540	1032.5617	-7.45	10 -	18	0	R.HGHLGFLPR.K
1079.5137	1078.5064	1078.5043	1.93	136 -	145	0	K.YAQDGAGIER.E
1096.5307	1095.5234	1095.5210	2.20	222 -	231	0	K.GHGFEGVTHR.W
1410.8053	1409.7981	1409.7918	4.42	37 -	49	0	R.SKPVALTSFLGYK.A
1666.8850	1665.8777	1665.8686	5.48	175 -	190	0	K.AHLAEIQLNGGSISEK.V
1714.8876	1713.8804	1713.8574	13.4	100 -	114	0	R.SLTTVWAEHLSDEVK.R
1716.8752	1715.8680	1715.8988	-18.00	50 -	65	1	K.AGMTTIVRDLDRPGSK.F
1870.9867	1869.9794	1869.9585	11.2	100 -	115	1	R.SLTTVWAEHLSDEVKR.R

No match to: 646.3642, 807.4072, 818.4323, 886.4792, 897.4088, 995.5017, 1065.5244, 1157.6073, 1159.5882, 1179.6046, 1234.6902, 1266.6573, 1307.6856, 1383.6930, 1393.7383, 1434.7806, 1475.7813, 1493.7536, 1497.8145, 1534.9156, 1557.7798, 1578.8201, 1638.8194, 1707.8196, 1741.8804, 1821.9360, 1837.9672, 1846.8948, 1855.0084, 2283.1744, 2705.1716, 2808.3529, 3264.5027, 3312.3126

Rps26

Mass-spectrum:

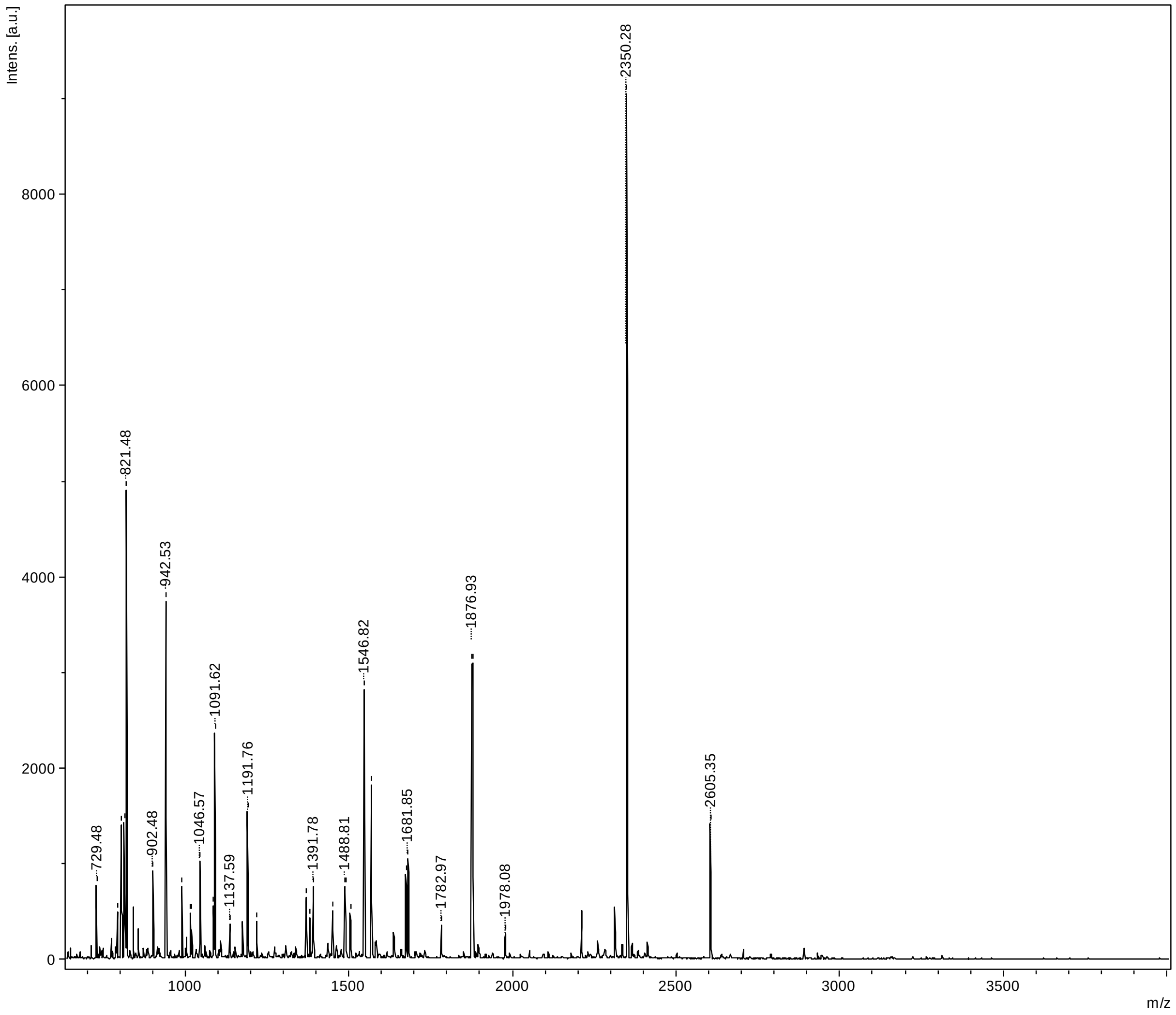

Identification:

gi|6320978 Mass: 13438 Score: 66 Expect: 0.012 Matches: 6

ribosomal 40S subunit protein S26B [Saccharomyces cerevisiae S288c]

Observed	Mr(expt)	Mr(calc)	ppm	Start	End	Miss	Peptide
792.4864	791.4792	791.4766	3.22	16 -	22	0	R.GHVKPVR.C
821.4820	820.4747	820.4668	9.67	96 -	102	0	R.APPQRPR.F
942.5341	941.5268	941.5294	-2.79	43 -	51	0	R.NIVEAAAVR.D
1091.6182	1090.6109	1090.6108	0.12	94 -	102	1	K.NRAPPQRPR.F
1681.8467	1680.8395	1680.8246	8.83	52 -	66	0	R.DLSEASVYPEYALPK.T
2605.3516	2604.3443	2604.3435	0.33	43 -	66	1	R.NIVEAAAVRDLSEASVYPEYALPK.T

No match to: 729.4757, 804.2801, 815.4336, 902.4818, 991.5648, 1016.5624, 1020.5810, 1046.5743, 1087.4978, 1137.5905, 1191.7608, 1219.7193, 1370.6568, 1381.8644, 1391.7792, 1451.8177, 1488.8089, 1490.8027, 1505.7911, 1546.8223, 1569.7700, 1674.9161, 1782.9732, 1876.9283, 1878.9926, 1978.0804, 2350.2830

Sup35

Mass-spectrum:

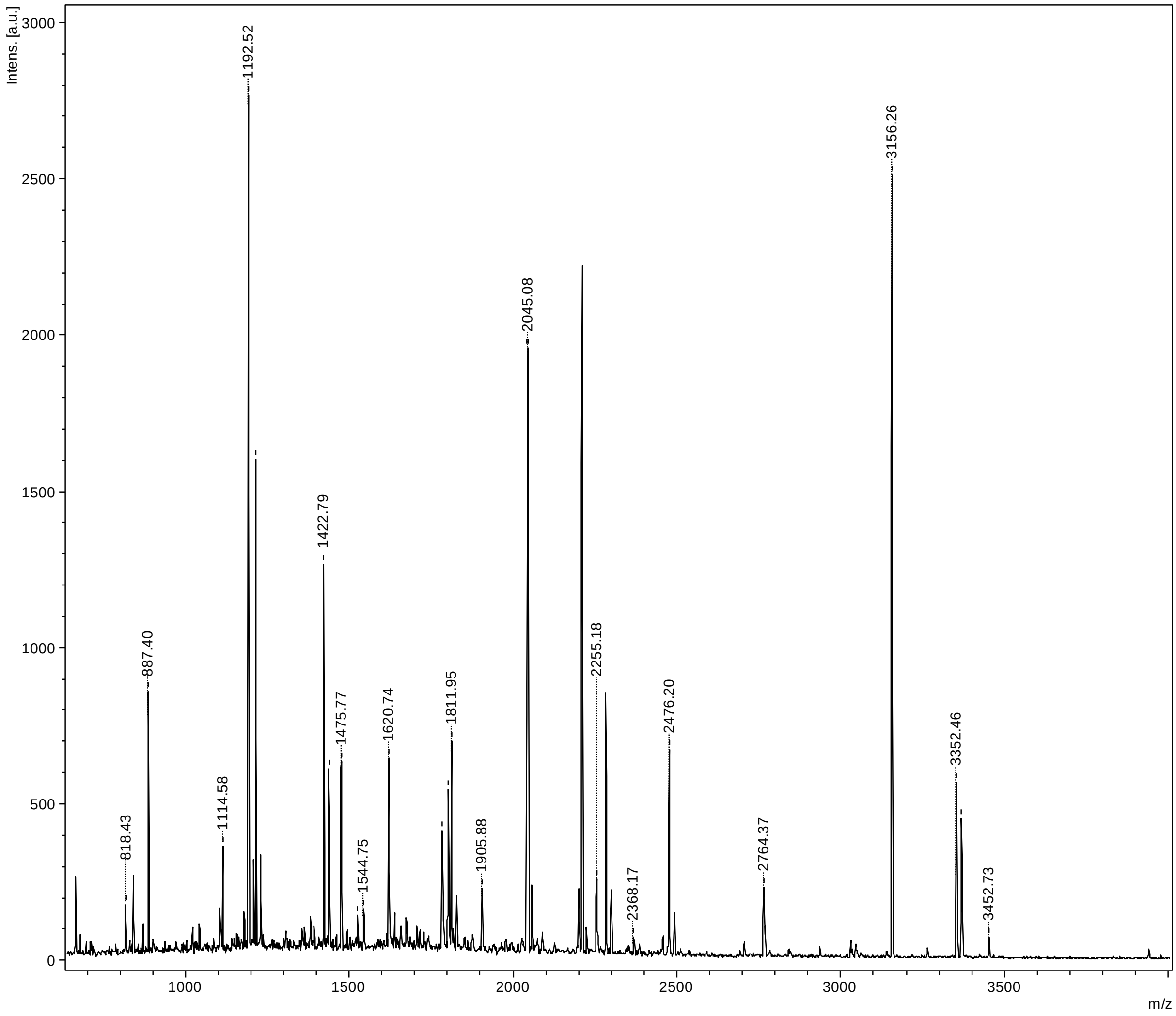

Identification:

gi|380005367 **Mass:** 76535 **Score:** **143** **Expect:** 1.7e-07 **Matches:** 15

Sup35 [Saccharomyces cerevisiae]

Observed	Mr(expt)	Mr(calc)	ppm	Start	End	Miss	Peptide
887.4042	886.3970	886.4072	-11.58	331	337	0	K.AYFETEK.R
1114.5770	1113.5697	1113.5818	-10.87	340	349	0	R.YTILDAPGHK.M
1192.5249	1191.5176	1191.5230	-4.56	408	417	0	K.MDDPTVNWSK.E
1215.5733	1214.5660	1214.5567	7.62	373	382	1	R.KGEYETGFER.G
1422.7948	1421.7875	1421.7853	1.56	485	497	0	R.HINAPFMLPIAAK.M
1438.7819	1437.7746	1437.7802	-3.89	485	497	0	R.HINAPFMLPIAAK.M + Oxidation (M)
1784.8919	1783.8846	1783.8815	1.77	440	456	0	K.TDVVFMPVSGYSGANLK.D
1803.9176	1802.9104	1802.8938	9.17	557	573	0	K.GVEEEDISPGFVLTSPK.N
1811.9488	1810.9415	1810.9247	9.28	274	290	1	K.STMGGNLLYLTGSVDKR.T
2041.9728	2040.9655	2040.9363	14.3	305	320	1	R.QGWYLSWVMDTNKEER.N
2045.0780	2044.0708	2044.0728	-1.01	555	573	1	R.IKGVEEEDISPGFVLTSPK.N
2476.1986	2475.1913	2475.2104	-7.73	440	462	1	K.TDVVFMPVSGYSGANLKDHVDPK.E
2764.3709	2763.3637	2763.3789	-5.52	642	665	0	K.VIAVLETEAPVCVETYQDYPQLGR.F + Propionamide (C)
3352.4607	3351.4535	3351.5003	-13.98	103	131	0	K.NFNYNNNLQGYQAGFQPQSQGMSLNDFQK.Q
3368.4514	3367.4441	3367.4952	-15.19	103	131	0	K.NFNYNNNLQGYQAGFQPQSQGMSLNDFQK.Q + Oxidation (M)

No match to: 818.4326, 1475.7732, 1620.7421, 1905.8754, 2255.1809, 2283.1695, 3156.2596, 3452.7316

Tef2

Mass-spectrum:

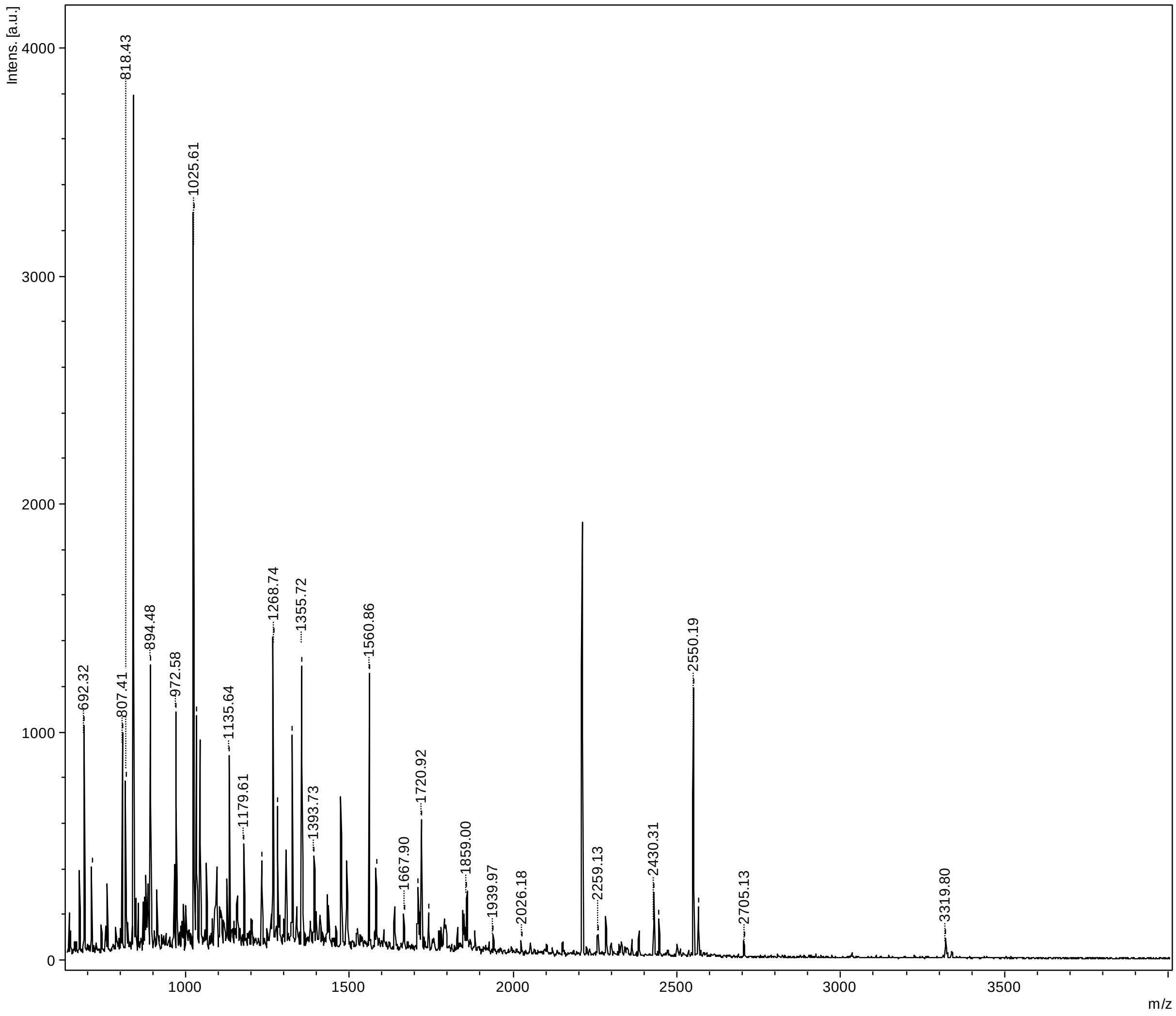

Identification:

gi|32563240 Mass: 41199 **Score:** 129 **Expect:** 4.2e-06 **Matches:** 13

translation elongation factor 1-alpha, partial [Saccharomyces cerevisiae]

Observed	Mr(expt)	Mr(calc)	ppm	Start	End	Miss	Peptide
894.4768	893.4696	893.4647	5.44	35 -	41	0	K.YAWVLDK.L
1025.6078	1024.6005	1024.6030	-2.39	233 -	243	0	K.IGGIGTVPVGR.V
1033.5497	1032.5424	1032.5604	-17.44	1 -	9	0	-.TTTGHLIYK.C
1135.6393	1134.6320	1134.6437	-10.29	35 -	43	1	K.YAWVLDKLK.A
1326.7641	1325.7568	1325.7456	8.51	114 -	125	0	R.EHALLAFTLGVR.Q
1355.7207	1354.7134	1354.6994	10.4	64 -	75	0	K.YQVTVIDAPGHR.D
1582.8612	1581.8540	1581.8402	8.68	144 -	156	1	R.FQEIVKETSNFIK.K
1858.9964	1857.9891	1857.9737	8.29	64 -	79	1	K.YQVTVIDAPGHRDFIK.N
2430.3131	2429.3059	2429.3240	-7.47	244 -	267	0	R.VETGVIKPGMVVTFAPAGVTTEVK.S
2446.3118	2445.3045	2445.3189	-5.89	244 -	267	0	R.VETGVIKPGMVVTFAPAGVTTEVK.S + Oxidation (M)
2550.1884	2549.1811	2549.1969	-6.19	268 -	290	0	K.SVEMHHEQLEQGVPGDNVGFNVK.N
2566.1879	2565.1806	2565.1918	-4.38	268 -	290	0	K.SVEMHHEQLEQGVPGDNVGFNVK.N + Oxidation (M)
3319.7959	3318.7886	3318.8187	-9.06	204 -	232	1	K.TLLEAIDAIEQPSRPTDKPLRLPLQDVYK.I

No match to: 692.3229, 715.0093, 807.4135, 818.4307, 972.5791, 1045.5619, 1179.6067, 1234.6817, 1268.7374, 1282.7593, 1307.6861, 1393.7284, 1475.7783, 1493.7553, 1560.8640, 1638.8665, 1710.9555, 1720.9235, 1741.8722, 1939.9736, 2026.1837, 2259.1309, 2705.1257
