# Supplementary material for: Proteomic Screening for Amyloid Proteins
Source: PLoS One. 2014 Dec 30;9(12):e116003. doi: 10.1371/journal.pone.0116003 (PMC4280166; doi:10.1371/journal.pone.0116003)
Supplement: S5 Table — Identification data of the proteins listed in Table 5. (PDF) [file pone.0116003.s005.pdf]

Table S5. Identification data of the proteins listed in Table 5.

Bmh2

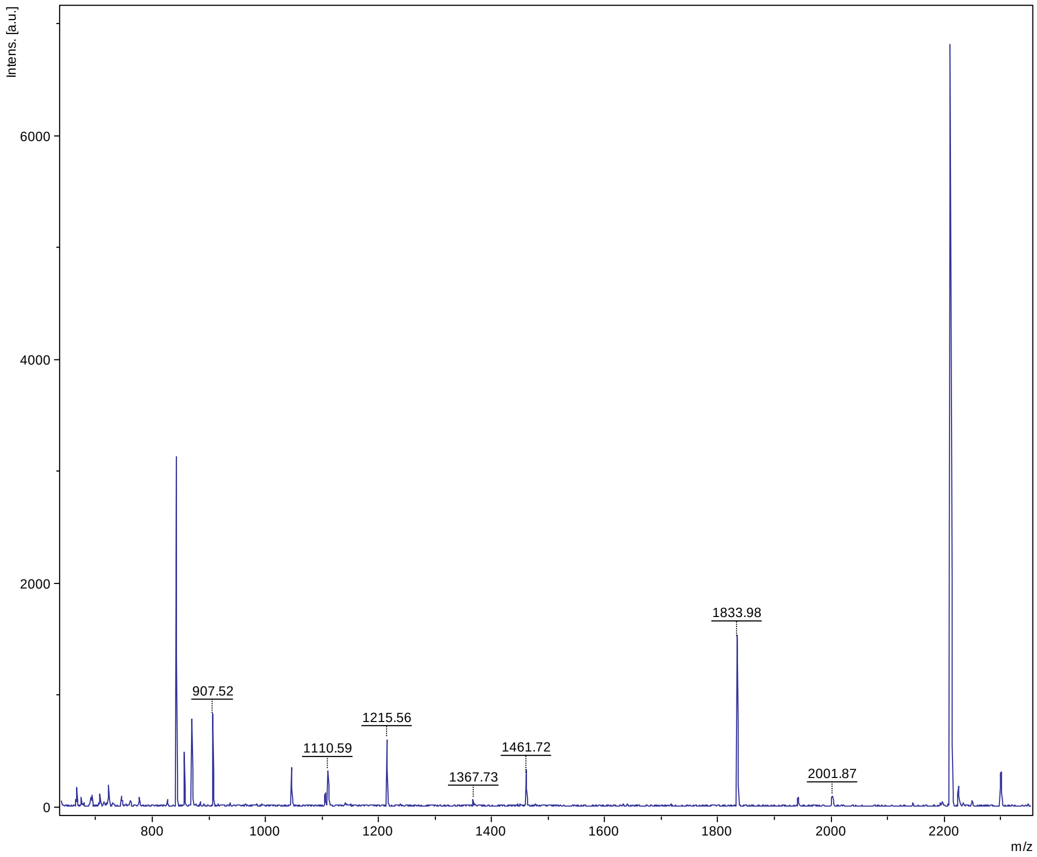

gi|398365597 **Mass:** 31042 **Score: 119 Expect:** 3e-05 **Matches:** 7

Bmh2p [Saccharomyces cerevisiae S288c]

Observed	Mr(expt)	Mr(calc)	ppm	Start		End	Miss	Peptide
907.5216	906.5144	906.5174	-3.40	44	-	51	0	R.NLLSVAYK.N
1110.5938	1109.5865	1109.5829	3.23	77	-	85	0	K.SEHQVELIR.S
1215.5612	1214.5539	1214.5567	-2.32	133	-	143	0	R.YLAEFSSGDAR.E
1367.7297	1366.7224	1366.7204	1.42	75	-	85	1	K.EKSEHQVELIR.S
1461.7239	1460.7166	1460.7107	4.05	30	-	43	0	K.AVASSGQELSVEER.N
1833.9838	1832.9765	1832.9632	7.28	156	-	172	0	K.TASEIATTELPPTHPIR.L
2001.8733	2000.8660	2000.8819	-7.93	14	-	29	1	K.LAEQAERYEEMVENMK.A + 2 Oxidation (M)

No match to: 1940.9135

Def1

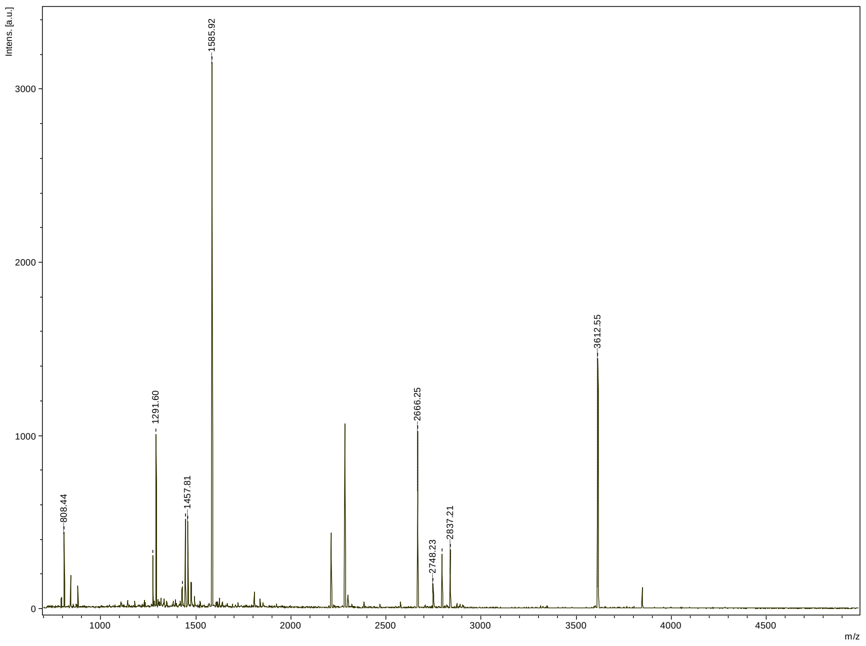

gi|6322796 **Mass:** 83923 **Score: 106 Expect:** 0.00057 **Matches:** 9

Def1p [Saccharomyces cerevisiae S288c]

Observed	Mr(expt)	Mr(calc)	ppm	Start		End	Miss	Peptide
1273.5876	1272.5803	1272.5582	17.4	104	-	115	0	K.SSNNSNSFTSTK.H
1291.6026	1290.5953	1290.5701	19.6	116	-	126	0	K.HNSSNNYTQAR.N
1428.8277	1427.8204	1427.7959	17.2	241	-	253	1	K.KMSWAAIATPKPK.A
1444.8256	1443.8183	1443.7908	19.1	241	-	253	1	K.KMSWAAIATPKPK.A + Oxidation (M)
1457.8148	1456.8076	1456.7773	20.8	258	-	270	1	K.TESPLENVAELKK.E
2666.2499	2665.2426	2665.2408	0.68	82	-	103	0	K.EQQHSYVPQQHLPNPEDDITYK.S
2794.3467	2793.3394	2793.3358	1.29	81	-	103	1	K.KEQQHSYVPQQHLPNPEDDITYK.S
2837.2100	2836.2028	2836.2132	-3.67	172	-	196	0	K.HDVPQDSNDNNNEELEAQGQQAQEK.N
3612.5492	3611.5419	3611.5533	-3.17	631	-	661	0	K.NNYNYYQTQNGQEQQSPNQGVAQHSEDSQQK.Q

No match to: 808.4375, 1585.9177, 2748.2307

Pub1

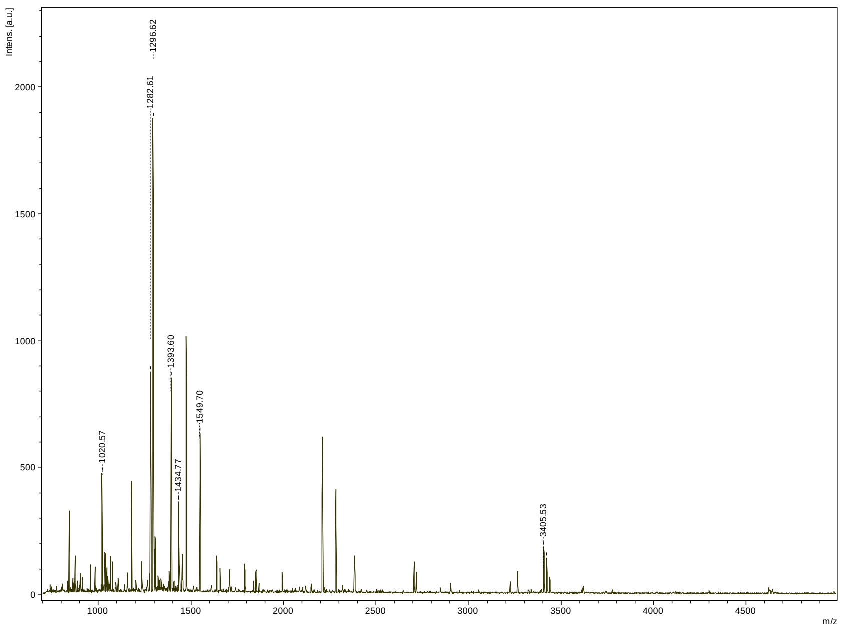

gi|172438 **Mass:** 47834 **Score:** 78 **Expect:** 0.41 **Matches:** 6

RNA-binding protein [Saccharomyces cerevisiae]

Observed	Mr(expt)	Mr(calc)	ppm	Start		End	Miss	Peptide
1020.5688	1019.5616	1019.5651	-3.51	76	-	84	0	R.VLYVGNLDK.A
1393.6021	1392.5948	1392.5766	13.1	242	-	252	0	R.DNNNNNNYQQR.R
1434.7750	1433.7677	1433.7667	0.72	93	-	105	0	K.QYFQVGGPIANIK.I
1549.6962	1548.6889	1548.6777	7.24	242	-	253	1	R.DNNNNNNYQQRR.N
3405.5309	3404.5236	3404.5150	2.54	204	-	234	0	R.GYGFVSFTSQDDAQNAMDSMQGQDLNGRPLR.I
3421.5172	3420.5100	3420.5099	0.01	204	-	234	0	R.GYGFVSFTSQDDAQNAMDSMQGQDLNGRPLR.I + Oxidation (M)

No match to: 1282.6066, 1296.6220

Rpn10

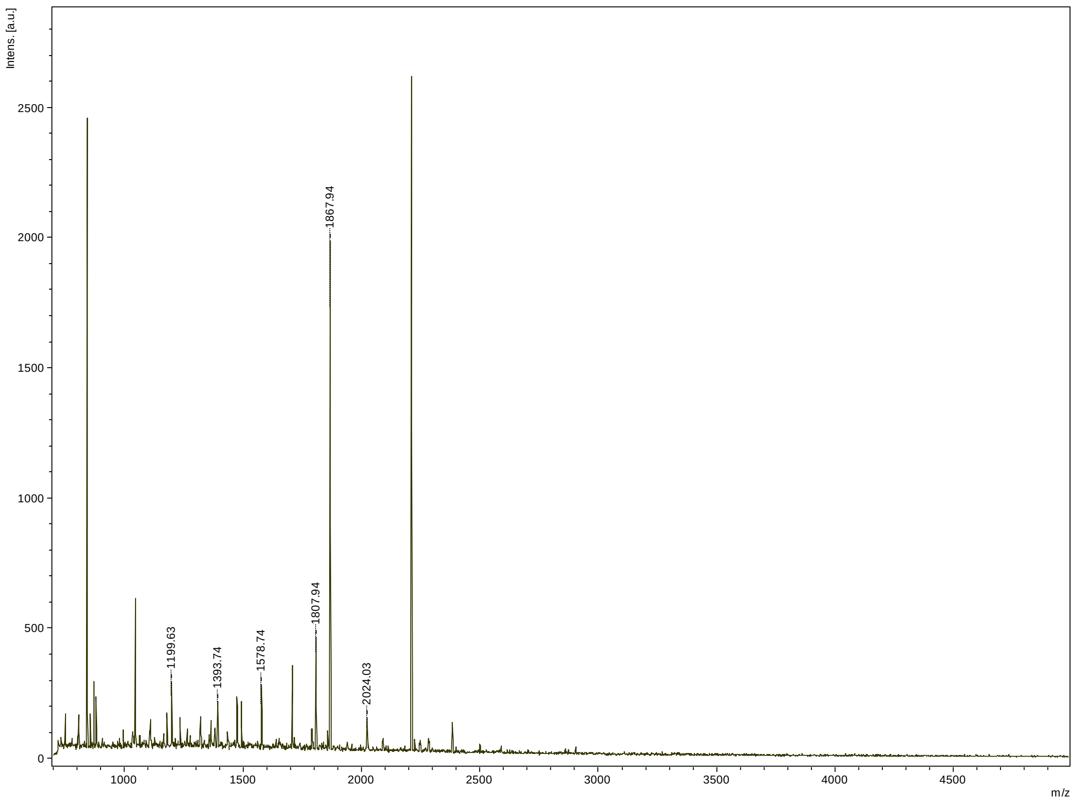

gi|398365027 **Mass:** 29729 **Score:** **120** **Expect:** 2.3e-05 **Matches:** 6

Rpn10p [Saccharomyces cerevisiae S288c]

Observed	Mr(expt)	Mr(calc)	ppm	Start		End	Miss	Peptide
1199.6343	1198.6270	1198.6234	2.99	61	-	71	0	R.VLSTFTAEFGK.I
1393.7411	1392.7338	1392.7071	19.1	110	-	122	0	R.IVAFVCSPISDSR.D
1578.7375	1577.7302	1577.7103	12.6	234	-	245	1	R.LSMEEEQQRQER.L + Oxidation (M)
1807.9444	1806.9371	1806.9363	0.43	2	-	17	0	M.VLEATVLVIDNSEYSR.N
1867.9352	1866.9279	1866.9184	5.12	42	-	60	0	R.NSNPENTVGLISGAGANPR.V
2024.0335	2023.0262	2023.0195	3.32	41	-	60	1	K.RNSNPENTVGLISGAGANPR.V

Sgt2

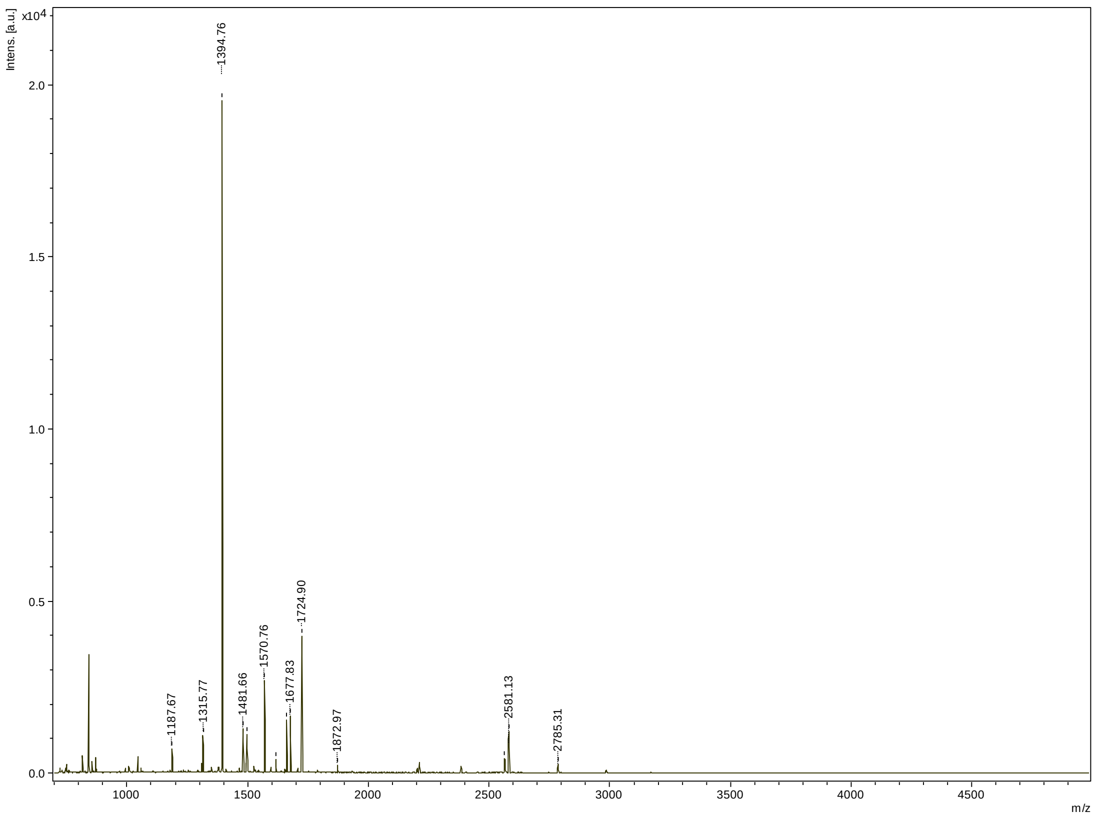

gi|6324580 **Mass:** 37195 **Score:** **133** **Expect:** 1.1e-06 **Matches:** 11

Sgt2p [Saccharomyces cerevisiae S288c]

Observed	Mr(expt)	Mr(calc)	ppm	Start	End	Miss	Peptide
1187.6736	1186.6663	1186.6557	8.91	218 -	227	1	K.KVEQSLNLEK.T
1394.7570	1393.7497	1393.7354	10.3	131 -	142	0	K.VLPTNAIYYANR.A
1481.6616	1480.6543	1480.6472	4.79	276 -	288	0	K.MMSNPGAMQNIQK.M + 2 Oxidation (M)
1497.6569	1496.6496	1496.6422	4.96	276 -	288	0	K.MMSNPGAMQNIQK.M + 3 Oxidation (M)
1570.7581	1569.7508	1569.7311	12.6	158 -	171	0	K.DAESAISIDPSYFR.G
1617.8412	1616.8339	1616.8158	11.2	143 -	157	1	R.AAAHSSLKEYDQAVK.D
1661.8358	1660.8285	1660.8090	11.8	196 -	210	1	K.VLDIEGDNATEAMKR.D
1677.8299	1676.8226	1676.8039	11.1	196 -	210	1	K.VLDIEGDNATEAMKR.D + Oxidation (M)
1724.8991	1723.8918	1723.8780	7.98	181 -	195	1	K.YAQGKPEEALEAYKK.V
1872.9711	1871.9639	1871.9489	7.97	58 -	74	1	K.SEFKGQHLADILNSASR.V
2564.1647	2563.1574	2563.1795	-8.61	297 -	321	0	R.QMAEGFASGGGTPNLSDLMNNPALR.N + Oxidation (M)

No match to: 1315.7682, 2581.1346, 2785.3130

Sup35

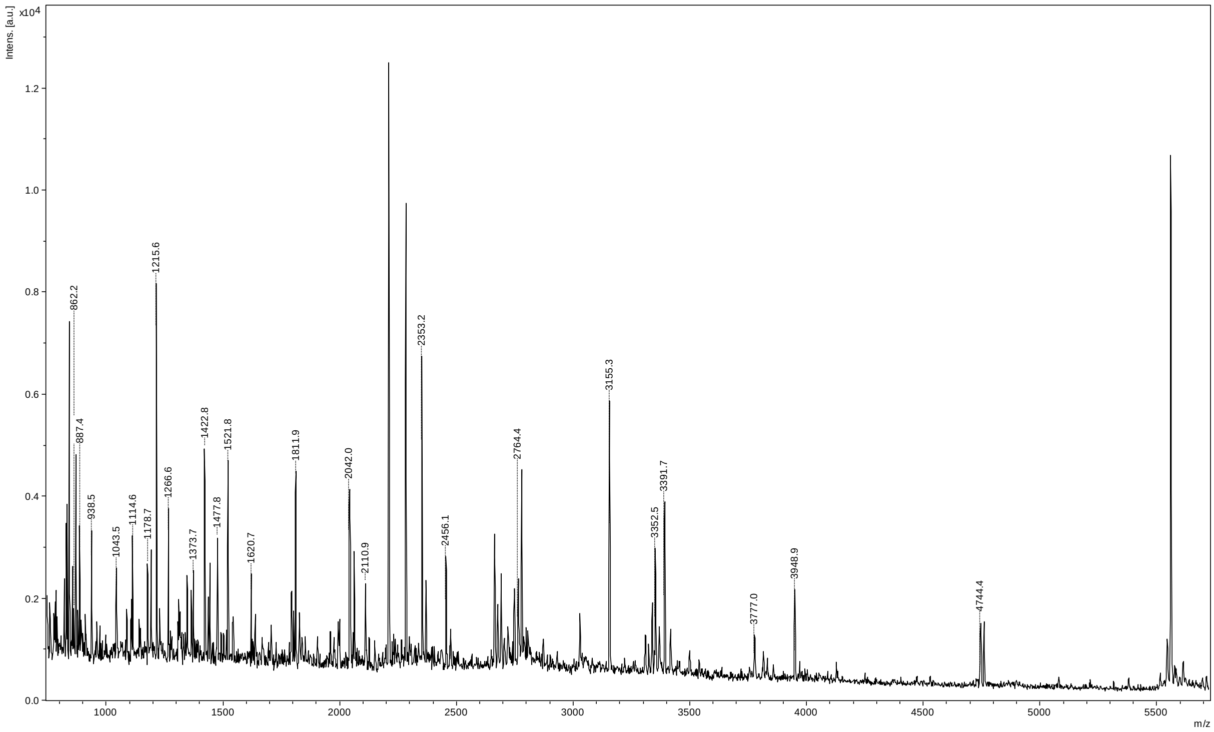

gi|16304381 **Mass:** 48307 **Score:** **108** **Expect:** 0.00054 **Matches:** 16

Sup35p [Saccharomyces cerevisiae]

Observed	Mr(expt)	Mr(calc)	ppm	Start		End	Miss	Peptide
887.4207	886.4134	886.4072	6.99	329	-	335	0	K.AYFETEK.R
938.4867	937.4794	937.4869	-7.94	289	-	295	1	R.TIEKYER.E
1043.5104	1042.5031	1042.5083	-4.98	329	-	336	1	K.AYFETEKR.R
1089.5063	1088.4990	1088.5350	-33.00	159	-	168	0	K.VDTKPAESDK.K
1114.5823	1113.5750	1113.5818	-6.17	338	-	347	0	R.YTILDAPGHK.M
1178.6672	1177.6599	1177.6740	-11.98	141	-	151	1	K.TLKLVSSSCIK.L
1192.5354	1191.5281	1191.5230	4.26	406	-	415	0	K.MDDPTVNWSK.E
1215.5698	1214.5626	1214.5567	4.79	371	-	380	1	R.KGEYETGFER.G
1422.7893	1421.7820	1421.7626	13.6	288	-	298	3	K.RTIEKYEREAK.D
1811.9280	1810.9207	1810.9247	-2.20	272	-	288	1	K.STMGGNLLYLTGSVDKR.T
2041.9514	2040.9441	2040.9363	3.82	303	-	318	1	R.QGWYLSWVMDTNKEER.N
2353.1922	2352.1849	2352.1817	1.36	348	-	370	0	K.MYVSEMIGGASQADVGVLVISAR.K
2369.1788	2368.1716	2368.1767	-2.16	348	-	370	0	K.MYVSEMIGGASQADVGVLVISAR.K + Oxidation (M)
2456.1294	2455.1221	2455.1226	-0.20	303	-	322	2	R.QGWYLSWVMDTNKEERNDGK.T
3352.4929	3351.4856	3351.5003	-4.39	101	-	129	0	K.NFNYNNNLQGYQAGFQPQSQGMSLNDFQK.Q
3776.9762	3775.9689	3775.9502	4.96	336	-	370	3	K.RRYTILDAPGHKMYVSEMIGGASQADVGVLVISAR.K + Oxidation (M)

No match to: 862.2412, 1266.5848, 1347.6540, 1366.6686, 1373.6664, 1438.7753, 1444.7279, 1477.7539, 1520.8393, 1521.8022, 1620.7159, 1803.8891, 2062.9518, 2110.9431, 2764.3759, 3155.3018, 3391.7431, 3948.9291, 4744.3942, 4758.4237
